# Occupational brucellosis among livestock farmers in Northeastern Thailand: A cross-sectional study of knowledge, attitudes, and practices from a One Health perspective

**DOI:** 10.1371/journal.pntd.0014788

**Published:** 2026-10-27

**Authors:** Savek Kiatsomphob, Karunasinee Suphaphiphat, Bopit Puyati, Kan Kledmanee

**Affiliations:** 1 Department of Animal Science, Faculty of Agriculture, Kasetsart University, Bangkok, Thailand; 2 Faculty of Veterinary Medicine, Chiang Mai University, Chiang Mai, Thailand; 3 Buriram Provincial Livestock Office, Buriram, Thailand; 4 Department of Epidemiology, Faculty of Public Health, Mahidol University, Bangkok, Thailand; University of Queensland School of Veterinary Science, AUSTRALIA

## Abstract

Brucellosis remains a significant occupational zoonotic disease among livestock farmers, with substantial implications for animal health, worker safety, and public health in endemic regions. Effective control at the livestock-human interface depends on understanding farmers’ knowledge, attitudes, and practices (KAP) regarding disease prevention. This study aimed to assess determinants of occupational brucellosis prevention behaviors among ruminant farmers in Northeastern Thailand through comprehensive KAP analysis to inform targeted One Health strategies. A cross-sectional survey was conducted from October 2024 to July 2025 across three provinces (Nakhon Ratchasima, Buriram, and Surin) in lower Northeastern Thailand. Structured questionnaires were administered to 385 livestock farmers managing ruminants (beef cattle, dairy cattle, buffalo, goats, or sheep). Knowledge, attitude, and practice domains were scored and analyzed using logistic regression to identify behavioral determinants. Although 65.5% of farmers demonstrated satisfactory knowledge, only 20.0% reported comprehensive favorable preventive practices across personal and farm-level measures. Notably, farmers exhibited selective engagement with prevention: while all avoided raw milk and placental product consumption, only 38.7% used gloves during high-risk activities compared to 61.6% wearing boots. Multivariate analysis identified farm certification, including Good Farm Management as shown by adjusted Odds Ratio (aOR) 2.60 (95% CI: 1.06-6.39), and brucellosis-free certification as shown by aOR 5.35 (95% CI: 1.63–17.54). Awareness of having heard regarding the scientific term “brucellosis” was associated with favorable practices (aOR 2.74, 95% CI: 1.00–7.46). In conclusion, knowledge alone was insufficient to drive consistent preventive behaviors, instead, farm certification, targeted education on transmission routes, and perceived occupational susceptibility emerged as key drivers of practice adoption. Our findings highlight the necessity for integrated interventions combining domain-specific training, formal disease alerts, and structural supports such as certification programs to enhance occupational safety and brucellosis control aligned with One Health principles in Thailand.

## Author summary

In Northeastern Thailand, we conducted a field-based knowledge, attitudes, and practices (KAP) survey among ruminant (cattle, buffalo, goat, sheep) farmers. Our results showed that farms with official certifications, such as Good Farm Management (GFM), Good Agricultural Practice (GAP), or brucellosis‑free farm status, were more likely to report good protective behaviors against brucellosis. We also found that what people know matters in a very specific way: understanding how brucellosis is transmitted was more strongly linked to protective behavior than simply having heard of the disease. At the same time, farmers did not use all protective equipment equally. They commonly wore boots during risky activities like assisting with birth, but were much less likely to wear gloves, which are crucial for preventing infection when handling birth materials. In this setting, farmers rarely drink raw milk, so the main risk of brucellosis comes from direct contact with infected animals and their secretions during everyday farm work, shifting the focus of prevention from food safety to occupational biosafety during high-risk animal contact.

## Introduction

Brucellosis constitutes a globally significant occupational zoonotic disease, exerting substantial impact on animal health, worker safety, and public health, especially in endemic regions [1]. Globally, the disease is heavily underreported; current epidemiological models estimate the true annual burden at 1.6 to 2.1 million new human cases, concentrated primarily in Asia and Africa [2]. Furthermore, meta-analytic data shows a pooled worldwide human seroprevalence of approximately 15.5%, which is significantly higher in males (19.1%) than in females (14.0%) due to occupational livestock contact [2]. This infection poses a persistent threat to the livelihoods of farmers and rural communities worldwide. Effective control therefore demands a comprehensive understanding of local risk factors and preventive behaviors.

In Thailand, brucellosis has re-emerged as a significant zoonotic disease since 2003 [3,4] under the Thailand Communicable Diseases Act B.E. 2558 [5], establishing itself as endemic within ruminant populations [6,7], with reported herd-level seroprevalence rates reaching 5.1% in beef cattle, 4.9% in dairy cattle, 6.1% in buffaloes, and 6.9% in small ruminants [7]. National surveillance confirms a steady accumulation of human cases over subsequent decades, cementing brucellosis as an enduring public health challenge. Local investigations underscore that human infections concentrate within specific live-animal trading loops; for instance, contact within intensive goat farm and transport networks has yielded localized outbreaks with a 1.8% attack rate among exposed keepers. Furthermore, transmission extends beyond farm laborers into the broader rural community, with documented seropositivity discovered even within vulnerable clinical cohorts, such as pregnant women experiencing adverse pregnancy outcomes [8–10].

The predominant smallholder mixed farming systems, where livestock are often closely integrated with crop production, create an environment highly conducive to cross-species transmission [11]. In Thailand, small ruminants have been a primary focus of brucellosis control following the re-emergence of human cases linked to goat outbreaks and manual handling of aborted materials without protection [12]. National surveillance demonstrates persistent *Brucella* spp. infection in livestock, consistent with a complex multi-species transmission ecology [13]. Specifically, *B. melitensis* remains prevalent in small ruminants, with an individual-level seroprevalence of 0.72% and a herd-level rate of 9.31% [5]. Concurrently, *B. abortus* circulation is documented across large ruminants, with national herd prevalence rates reaching 5.1% in beef cattle, 4.9% in dairy cattle, and 6.1% in buffaloes [14]. Shared pasturing and free-ranging management facilitate cross-species contamination among co-mingled cattle, buffaloes, sheep, and goats [11]. This dynamic is further illustrated in Sing Buri Province, where communal grazing and the retention of clinically suspected animals were identified as primary risk factors for herd-level seropositivity in goats [15].

Evidence from a meta-analysis and occupational studies indicate that adequate biosecurity and consistent personal protective equipment (PPE) use are associated with lower seroprevalence of *Brucella* and occupational exposure [16–18]. To strengthen these behaviors, the Knowledge, Attitudes, and Practices (KAP) framework is widely utilized to understand and improve protective behavior in high-risk groups. In Thailand, prior KAP studies have remained within single-species production system, focusing either on small-ruminant (goat) farmers [19] or exclusively on large ruminant (dairy cattle) holders [20]. Existing evidence therefore lacks an examination of mixed-ruminant systems or broader structural determinants that may shape preventive choices in interconnected multi-species environments.

Understanding these factors is paramount for developing effective disease management strategies, particularly from a holistic One Health perspective, addressing interconnected human, animal, and environmental health risks [21]. Therefore, this study aimed to assess the KAP concerning occupational brucellosis among ruminant livestock farmers in Northeastern Thailand. Through a comprehensive KAP analysis, this research sought to identify key behavioral determinants of disease prevention, thereby informing the development of targeted, evidence based One Health strategies for effective brucellosis control and improved occupational safety at the community level.

## Results

### Study population

A total of 428 farmers were approached through the DLD‑registered farmer network, of whom 415 agreed to participate. The final analytical sample comprised 385 farmers after excluding records with incomplete data. These participants were distributed across Nakhon Ratchasima (164/385, 42.6%), Buriram (143/385, 37.1%), and Surin (78/385, 20.3%) (Table 1; Fig 1).

**Table 1:** Example definitions and classification criteria for KAP domains.

Domains and definition		Criteria for positive response
***Knowledge***		
	Correctly identified susceptible hosts and animal-to-human transmission risk (Host range and zoonotic susceptibility)	Correct
	Correctly identified the transmission risks of brucellosis from direct contact and consumption of animal’s raw product (Transmission routes of the disease)	Correct
	Correctly identified animal clinical signs (Clinical manifestations in animal)	Correct
	Correctly identified common human symptoms (Clinical manifestations in human)	Correct
	Correctly identified using personal protective equipment (PPE) as a prevention measure (Personal-level prevention)	Correct
	Correctly identified hygiene (hand washing) and disinfection as prevention measures (Personal-level prevention)	Correct
	Correctly identified the key farm biosecurity measures (new animal quarantine, sick animal isolation, and carcass disposal) as prevention practices (Farm-level prevention)	Correct
	Overall knowledge score	Satisfying if correctness is not less than 70% (i.e., ≥18 out of 26 items)
***Attitude***		
	**Perceived occupational susceptibility to brucellosis**	
	*Host range and zoonotic susceptibility*	
	Ruminant farmers have a higher risk of contracting brucellosis than people in other occupations.	“Strongly agree” and “agree”
	People in non-animal-related professions do not have the same risk level of contracting brucellosis as those who work directly with animals.	“Strongly agree” and “agree”
	Overall perceived susceptibility attitude, positive	Positive, when consistent favorable (“strongly agree” or “agree”) responses
	**Perceived disease impact and severity**	
	*Disease impact and severity*	
	Brucellosis has an impact on the economy and health of both humans and animals.	“Strongly agree” and “agree”
	Brucellosis is a disease that needs to be prioritized for control and prevention.	“Strongly agree” and “agree”
	Overall perceived disease impact and severity, positive	Positive, when consistent favorable (“strongly agree” or “agree”) responses
	**Attitude toward prevention and control**	
	*Recognition of risky transmission route*	
	It is necessary to boil milk before consumption to reduce the risk of brucellosis.	“Strongly agree” and “agree”
	*Personal protective measures and hygiene*	
	Washing hands after contacting animals is not troublesome.	“Strongly agree” and “agree”
	Using personal protective equipment, such as boots and gloves, is necessary when working with farm animals.	“Strongly agree” and “agree”
	Overall personal prevention attitude, positive	Positive, when consistent favorable (“strongly agree” or “agree”) responses
	*Farm biosecurity*	
	Using disinfectant on the farm is necessary.	“Strongly agree” and “agree”
	New animals purchased or brought into the farm may carry brucellosis and potentially transmit it to the existing herd.	“Strongly agree” and “agree”
	It is necessary to notify a veterinarian or Department of Livestock officers if farm animals are sick and suspected of having brucellosis.	“Strongly agree” and “agree”
	Overall farm-level prevention attitude, positive	Positive, when consistent favorable (“strongly agree” or “agree”) responses
	**Facilitators to animal brucellosis screening**	
	Testing animals on the farm for brucellosis is not a waste of labor.	“Strongly agree” and “agree”
	Farmers are willing to cooperate if free brucellosis testing services are provided.	“Strongly agree” and “agree”
	Overall facilitators attitude, positive	Positive, when consistent favorable (“strongly agree” or “agree”) responses
	Overall combined attitude toward occupational brucellosis, positive	Positive, when consistent favorable (“positive”) responses in all domains
***Practice***		
	*Daily farm operations*	
	Wearing boots is practiced while working on the farm.	“Always” and “often”
	*Post-activity hygiene*	
	Cleaning the body after farm work	“Always” and “often”
	Overall personal preventive practice and hygiene during daily farm activity, favorable	
	*Birth assistance management*	
	Wearing gloves is adopted when assisting with animal births.	“Always” and “often”
	Wearing boots is practiced when assisting with animal births. <	“Always” and “often”
	Overall personal preventive practice during birth assistance, favorable	
	**Farm biosecurity**	
	*Sanitation and disinfection practices*	
	Cleans and disinfects animal pens on the farm.	“Always” and “often”
	Disinfects vehicles that enter the farm	“Always” and “often”
	Overall combined preventive practice, favorable	Favorable, when consistent favorable (“always” and “often”) responses

**Fig 1:**
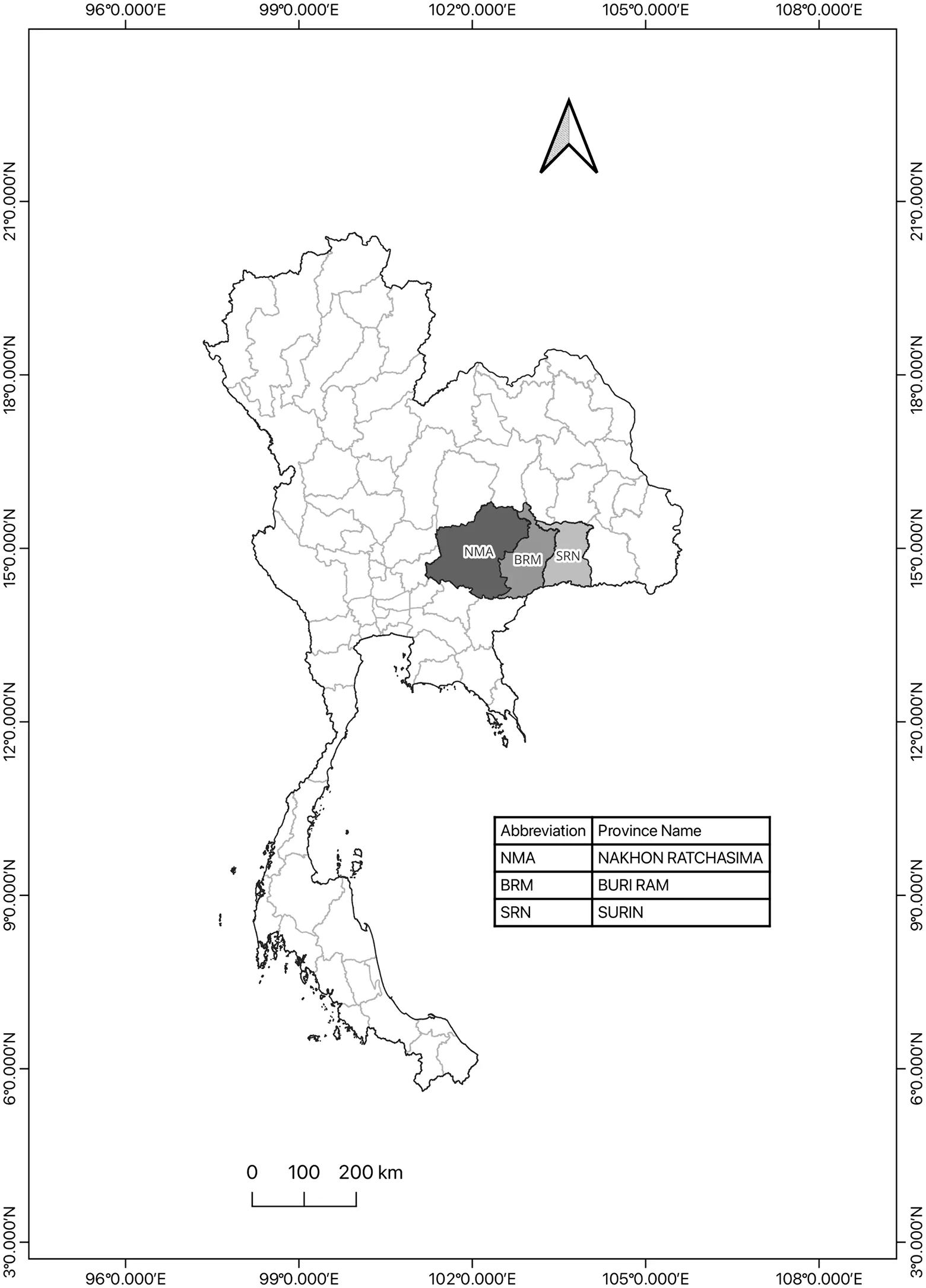
Study setting. This figure was generated with a shapefile from DIVA-GIS (https://diva-gis.org/) using the Open-Source Geospatial Foundation’s QGIS package (https://www.qgis.org/).

### Demographic and farm characteristics

The surveyed farmers 385 comprised nearly equal proportions of females (189/385, 49.1%) and males (196/385, 50.9%). The majority were aged 51–60 years (146/385, 37.9%), followed by 41–50 years (108/385, 28.1%). Older farmers (over 60 years) and those under 40 years represented in similar proportions (16.6%, 64/385 and 17.4% 67/385, respectively). Education levels varied, with 43.9% (169/385) having primary education or not completed, 40.5% (156/385) had completed secondary school, and 15.6% (60/385) held a diploma or higher education. Livestock farming experience varied among participants, which nearly one-thirds of participants (119/385, 30.9%) had 0–5 years of farming experience, followed by similar proportions with 6 – 10 years (113/385, 29.4%) and those with more than a decade (153/385, 39.7%) (**Table 2**).

**Table 2:** Sociodemographic characteristics and overall knowledge, attitude, and practice (KAP) levels regarding occupational brucellosis among ruminant farmers.

Farmer characteristics	Total n (%)	Knowledge^†^ (n = 385)			Attitude^†^ (n = 385)			Practice^†^ (n = 315)		
		Unsatisfying (n = 133) n (%)	Satisfying (n = 252) n (%)	p-value^a^	Negative (n = 380) n (%)	Positive (n = 5) n (%)	p-value^b^	Unfavorable (n = 252) n (%)	Favorable (n = 63) n (%)	p-value^b^
**Gender**				0.701			>0.999			0.272
Female	189 (49.1)	63 (47.4)	126 (50.0)		187 (49.2)	2 (40.0)		134 (53.2)	28 (44.4)	
Male	196 (50.9)	70 (52.6)	126 (50.0)		193 (50.8)	3 (60.0)		118 (46.8)	35 (55.6)	
**Age group (years)**				0.314			>0.999			0.992
≤ 40	67 (17.4)	23 (17.3)	44 (17.5)		66 (17.4)	1 (20.0)		43 (17.1)	11 (17.5)	
41 - 50	108 (28.1)	42 (31.6)	66 (26.2)		107 (28.2)	1 (20.0)		71 (28.2)	18 (28.6)	
51 - 60	146 (37.9)	52 (39.1)	94 (37.3)		144 (37.9)	2 (40.0)		97 (38.5)	23 (36.5)	
> 60	64 (16.6)	16 (12.0)	48 (19.0)		63 (16.6)	1 (20.0)		41 (16.3)	11 (17.5)	
**Education level**				0.346			>0.999			0.981
Primary education or not completed	169 (43.9)	65 (48.9)	104 (41.3)		167 (43.9)	2 (40.0)		105 (41.7)	26 (41.3)	
Secondary school	156 (40.5)	50 (37.6)	106 (42.1)		154 (40.5)	2 (40.0)		105 (41.7)	27 (42.9)	
Diploma or higher education	60 (15.6)	18 (13.5)	42 (16.7)		59 (15.5)	1 (20.0)		42 (16.7)	10 (15.9)	
**Primary household income source**				0.007			0.668			>0.999
Livestock farming with supplementary sources	256 (66.5)	76 (57.1)	180 (71.4)		252 (66.3)	4 (80.0)		162 (64.3)	41 (65.1)	
Livestock farming only	129 (33.5)	57 (42.9)	72 (28.6)		128 (33.7)	1 (20.0)		90 (35.7)	22 (34.9)	
**Years of livestock farming experience**				0.768			0.070			0.788
≤ 5	119 (30.9)	38 (28.6)	81 (32.1)		115 (30.3)	4 (80.0)		74 (29.4)	19 (30.2)	
6-10	113 (29.4)	40 (30.1)	73 (29.0)		113 (29.7)	0 (0.0)		83 (32.9)	18 (28.6)	
>10	153 (39.7)	55 (41.4)	98 (38.9)		152 (40.0)	1 (20.0)		95 (37.7)	26 (41.3)	
**Ruminant species owned**				0.010			0.043			0.366
Large ruminant (dairy cattle, beef cattle, buffalo)	258 (67.0)	101 (75.9)	157 (62.3)		257 (67.6)	1 (20.0)		162 (64.3)	36 (57.1)	
Small ruminant (goat, sheep)	127 (33.0)	32 (24.1)	95 (37.7)		123 (32.4)	4 (80.0)		90 (35.7)	27 (42.9)	
**Farm certification** ^**c**^										
***Good Farm Management; GFM***				0.324			0.795			< 0.001
None	271 (70.4)	97 (72.9)	174 (69.0)		266 (70.0)	5 (100.0)		190 (75.4)	32 (50.8)	
In process	64 (16.6)	17 (12.8)	47 (18.7)		64 (16.8)	0 (0.0)		34 (13.5)	18 (28.6)	
Certified	50 (13.0)	19 (14.3)	31 (12.3)		50 (13.2)	0 (0.0)		28 (11.1)	13 (20.6)	
***Good Agricultural Practice; GAP***				0.777			>0.999			0.041
None	291 (75.6)	98 (73.7)	193 (76.6)		286 (75.3)	5 (100.0)		187 (74.2)	41 (65.1)	
In process	49 (12.7)	19 (14.3)	30 (11.9)		49 (12.9)	0 (0.0)		29 (11.5)	15 (23.8)	
Certified	45 (11.7)	16 (12.0)	29 (11.5)		45 (11.8)	0 (0.0)		36 (14.3)	7 (11.1)	
***Brucellosis-free farm***				0.345			>0.999			< 0.001
None	315 (81.8)	114 (85.7)	201 (79.8)		310 (81.6)	5 (100.0)		213 (84.5)	39 (61.9)	
In process	50 (13.0)	14 (10.5)	36 (14.3)		50 (13.2)	0 (0.0)		28 (11.1)	16 (25.4)	
Certified	20 (5.2)	5 (3.8)	15 (6.0)		20 (5.3)	0 (0.0)		11 (4.4)	8 (12.7)	

a p-value from Chi-square test.

b p-value from the Fisher’s exact test.

c None: farms that had never initiated the certification process. In process: Farms currently applying for or actively participating in the certification program. Certified: Farms that had fully achieved and maintained official certification status from the National Department of Livestock Development (DLD).

† Knowledge and attitude outcomes are based on the full sample of farmers (N = 385). Practice outcomes are based on the predefined high-exposure subgroup of farmers who reported assisting animal births within the previous year (N = 315).

Most farmers managed large ruminant herds (258/385, 67.0%), with buffaloes (173/385, 44.9%) and beef cattle (116/385, 30.1%) being most common. Small ruminant farmers constituted 33.0% (127/385) with sheep ownership was extremely rare (11/385, 2.9%). Apart from ruminant livestock, non-ruminant species such as dogs or cats (212/385, 55.1%), poultry (97/385, 25.2%), and swine (26/385, 6.8%) were also observed. Farm certification by DLD was less prevalent, with 13.0% (50/385) holding Good Farm Management (GFM) certification,11.7% (45/385) Good Agricultural Practice (GAP) certification, and only 5.2% of farms (20/385) were certified as brucellosis-free farm. Further details of the distribution of KAP levels across farmers’ demographics and farm characteristics are presented in Table 2.

### KAP levels and specific practices

Among 385 farmers, heard of brucellosis by its standard term was distinctly low (115/385, 29.9%), contrasting with higher familiarity through local terminology (241/385, 62.6%). Approximately two-thirds (252/385, 65.5%) of farmers demonstrated satisfactory level of overall knowledge. Most farmers correctly identified the purpose of using PPE (363/385, 94.3%) and of hygiene and disinfection measures (335/385, 87.0%), while fewer recognized the key farm biosecurity measures (214/385, 55.6%). Only 96 farmers (24.9%) correctly identified facts regarding human brucellosis treatment. Notably, the majority of farmers (316/385, 82.1%) perceived notifying a veterinarian or DLD officer when brucellosis was suspected in their herd to be important, which was classified as positive preventive attitude toward farm biosecurity. Further details are reported in S1 Table and S2 Table.

Among the predefined high-exposure subgroup of 315 farmers who reported assisting animal births, wearing boots during birth assistance was the most commonly reported preventive practice (194/315, 61.6%), whereas wearing gloves was substantially less common (122/315, 38.7%). Comprehensive adherence to brucellosis preventive practice across person- and farm-level domains was infrequent (63/315, 20%) (S3 Table). The heatmap revealed that specific knowledge, particularly regarding transmission routes (49/63, 77.8% of favorable combined practice group), positive attitudes toward boiling milk for brucellosis prevention (48/63, 76.2%) or perceived disease impact and severity (44/63, 69.8%) were strongly linked with improved practice uptake across person- and farm-level subgroups (Fig 2). Additionally, key knowledge gaps were observed for host range and zoonotic susceptibility (103/315, 32.7%), as well as human clinical manifestation and treatment of human brucellosis (121/315, 38.4% and 79/315, 25.1%, respectively), with similar proportions across combined practice groups. Positive prevention attitudes towards personal protective measures and hygiene were associated with improved person-level preventive practice during daily farm activities (124/216, 57.4% among favorable practice group vs. 44/99, 44.4% among unfavorable group; p-value = 0.043). Further significance details are reported in S3 Table, S4 Table, and S5 Table.

**Fig 2:**
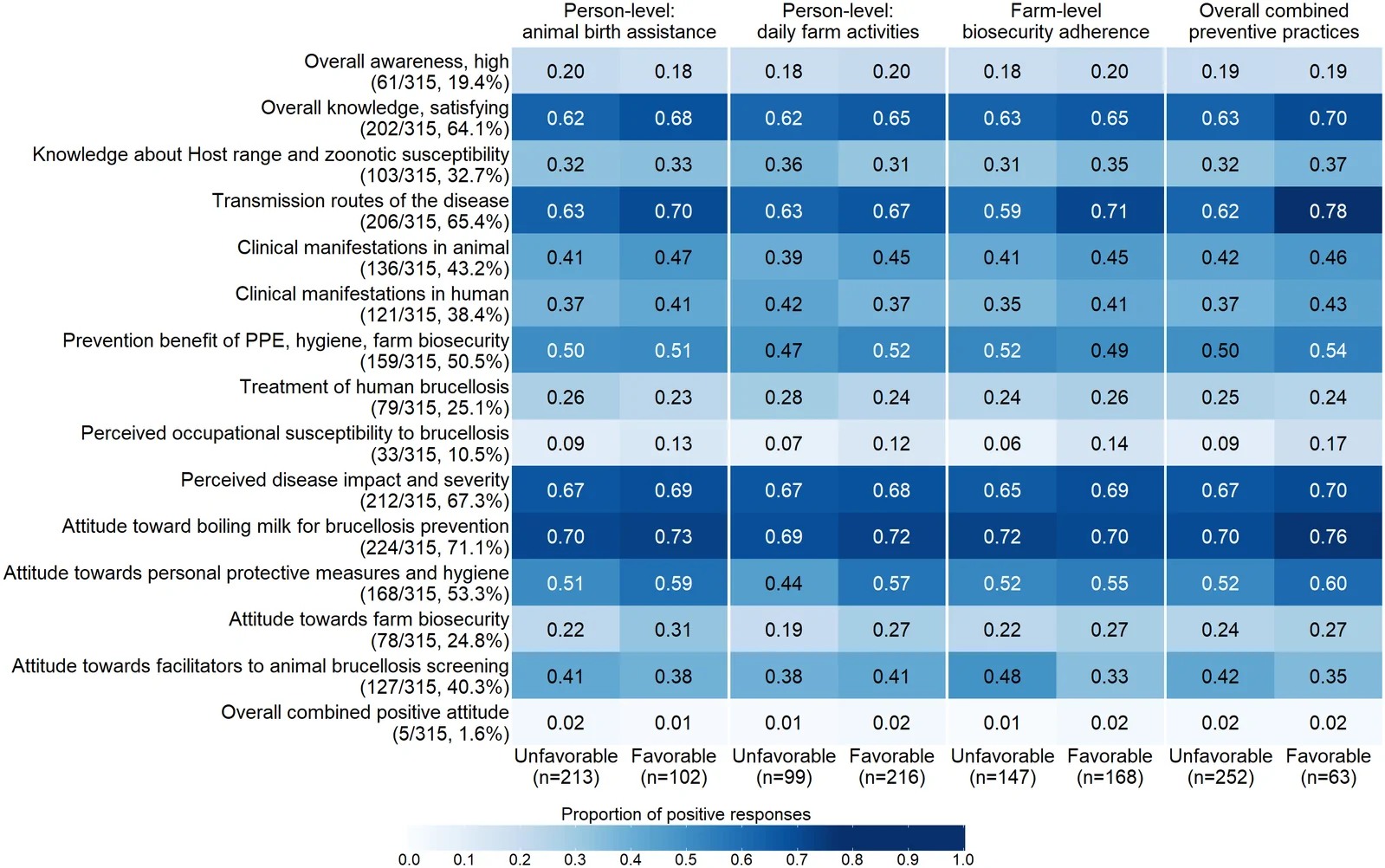
Heatmap of positive response proportions for occupational brucellosis-preventive practices across personal, farm-level, and combined domains among livestock farmers (N = 315). Proportions of positive responses to core knowledge, attitude, and practice (KAP) items related to occupational brucellosis prevention are shown for four preventive domains: personal-level practices during animal birth assistance, personal-level daily farm activities, farm-level biosecurity adherence, and overall combined preventive practices. Columns represent unfavorable and favorable practices, with sample sizes indicated. Color intensity reflects the proportion of positive responses per practice item, with higher values in darker blue. The right legend displays the scale of positive response proportions. Items cover awareness, knowledge, and attitudes toward preventive measures. See detailed findings in S3 Table, S4 Table, and S5 Table.

### Determinants of KAP and practices

Multiple logistic regression analysis was conducted to identify sociodemographic determinants associated with satisfactory knowledge, prevention-focused attitude, and combined preventive practices toward occupational brucellosis among ruminant farmers (Table 3). Farmers with diploma or higher education was associated with satisfactory knowledge (aOR 2.20, 95% CI: 1.00–4.81) and prevention-focused attitude (aOR 2.57, 95% CI: 1.05–6.26) compared to primary education or not completed. Moreover, farmers owning small ruminants (goats or sheep) had increased odds of satisfactory knowledge (aOR 1.93, 95% CI: 1.12–3.34) and attitude (aOR 1.76, 95% CI: 0.93–3.34) compared to those owning large ruminants. Financial reliance on livestock farming as a primary income source was negatively associated with satisfactory knowledge (aOR 0.54, 95% CI: 0.33–0.87) and with preventive -focused attitude (aOR 1.44, 95% CI: 0.80–2.60). Participation in GFM certification was strongly associated with improved combined preventive practices (aOR 2.46, 95% CI: 1.07-5.66), and certified brucellosis-free farms showed even greater association (aOR 5.35, 95% CI: 1.63-17.54). Farmers aged over 60 years also had higher odds of satisfactory knowledge compared with those aged 40 years or younger (aOR 2.65, 95% CI 1.06–6.62), whereas gender and years of farming experience showed no statistically significant associations with any KAP outcomes.

**Table 3:** Multivariable logistic regression analysis of sociodemographic factors of satisfactory knowledge, prevention-focused attitude, and combined preventive practices towards brucellosis among ruminant farmers.

Farmer characteristics	Knowledge^†^ (n=385)				Attitude^†^ (n=385)				Practice^†^ (n=315)			
	cOR (95%CI)	p-value^b^	aOR (95%CI)	p-value^b^	cOR (95%CI)	p-value^b^	aOR (95%CI)	p-value^b^	cOR (95%CI)	p-value^b^	aOR (95%CI)	p-value^b^
**Gender**												
Female	1		1		1		1		1		1	
Male	0.90 (0.59,1.37)	0.623	0.87 (0.55,1.36)	0.543	0.80 (0.47,1.35)	0.404	0.88 (0.5,1.54)	0.660	1.29 (0.75,2.24)	0.356	1.22 (0.67,2.24)	0.520
**Age group (years)**												
≤ 40	1		1		1		1		1		1	
41 - 50	0.82 (0.44,1.55)	0.544	1.01 (0.50,2.01)	0.984	0.64 (0.3,1.36)	0.243	0.84 (0.37,1.89)	0.672	1.02 (0.45,2.34)	0.958	1.71 (0.65,4.52)	0.278
51 - 60	0.94 (0.51,1.73)	0.855	1.40 (0.68,2.89)	0.368	0.66 (0.32,1.34)	0.247	1.05 (0.46,2.41)	0.903	0.99 (0.45,2.18)	0.973	1.56 (0.58,4.14)	0.376
> 60	1.57 (0.73,3.35)	0.245	2.65 (1.06,6.62)	0.036	0.46 (0.18,1.15)	0.097	0.89 (0.3,2.7)	0.844	1.06 (0.42,2.68)	0.896	1.60 (0.49,5.24)	0.436
**Education level**												
Primary education or not completed	1		1		1		1		1		1	
Secondary school	1.32 (0.84,2.09)	0.228	1.78 (1.03,3.09)	0.039	1.53 (0.83,2.79)	0.170	1.51 (0.75,3.04)	0.254	1.10 (0.61,2.00)	0.751	0.98 (0.46,2.07)	0.955
Diploma or higher education	1.46 (0.77,2.75)	0.243	2.20 (1.00,4.81)	0.049	2.43 (1.17,5.03)	0.017	2.57 (1.05,6.26)	0.038	1.08 (0.48,2.41)	0.860	0.85 (0.31,2.33)	0.746
**Primary household income source**												
Livestock farming with supplementary sources	1		1		1		1		1		1	
Livestock farming only	0.53 (0.34,0.83)	0.005	0.54 (0.33,0.87)	0.011	1.43 (0.83,2.46)	0.196	1.44 (0.80,2.60)	0.222	1.06 (0.6,1.88)	0.838	0.91 (0.47,1.76)	0.780
**Years of livestock farming experience**												
≤ 5	1		1		1		1		1		1	
6-10	0.86 (0.50,1.48)	0.577	1.04 (0.57,1.89)	0.894	1.32 (0.7,2.49)	0.396	1.26 (0.64,2.5)	0.502	0.91 (0.45,1.84)	0.784	0.95 (0.43,2.09)	0.891
>10	0.84 (0.50,1.39)	0.489	1.17 (0.64,2.16)	0.608	0.63 (0.32,1.22)	0.168	0.77 (0.35,1.68)	0.508	1.13 (0.59,2.19)	0.708	1.20 (0.53,2.73)	0.657
**Ruminant species owned**												
Large ruminant (dairy cattle, beef cattle, buffalo)	1		1		1		1		1		1	
Small ruminant (goat, sheep)	1.91 (1.19,3.06)	0.007	1.93 (1.12,3.34)	0.018	1.71 (1.00,2.94)	0.050	1.76 (0.93,3.34)	0.081	1.48 (0.85,2.59)	0.164	1.48 (0.73,2.97)	0.274
**Farm certification ^c^**												
***Good Farm Management; GFM***												
None	1		1		1		1		1		1	
In process	1.54 (0.84,2.83)	0.163	1.97 (0.93,4.2)	0.079	0.99 (0.48,2.04)	0.976	0.83 (0.35,2)	0.682	3.41 (1.73,6.70)	<0.001	2.46 (1.07,5.66)	0.034
Certified	0.91 (0.49,1.70)	0.765	0.84 (0.42,1.69)	0.624	1.05 (0.48,2.3)	0.910	0.72 (0.3,1.75)	0.469	3.05 (1.44,6.48)	0.004	2.60 (1.06,6.39)	0.037
***Good Agricultural Practice; GAP***												
None	1		1		1		1		1		1	
In process	0.80 (0.43,1.5)	0.488	0.64 (0.28,1.44)	0.280	1.33 (0.62,2.85)	0.462	1.47 (0.56,3.86)	0.435	2.75 (1.36,5.58)	0.005	1.19 (0.45,3.19)	0.727
Certified	0.92 (0.48,1.78)	0.804	1.2 (0.53,2.69)	0.665	1.48 (0.69,3.2)	0.315	1.43 (0.54,3.78)	0.474	1.03 (0.43,2.48)	0.941	0.49 (0.15,1.60)	0.235
***Brucellosis free farm***												
None	1		1		1		1		1		1	
In process	1.46 (0.75,2.82)	0.262	1.22 (0.52,2.89)	0.648	0.94 (0.42,2.12)	0.884	0.73 (0.25,2.11)	0.559	3.58 (1.77,7.21)	<0.001	2.54 (0.95,6.78)	0.062
Certified	1.70 (0.6,4.8)	0.316	1.72 (0.54,5.45)	0.356	2.12 (0.78,5.76)	0.142	1.74 (0.55,5.52)	0.346	4.55 (1.72,12.02)	0.002	5.35 (1.63,17.54)	0.006

cOR = crude odds ratio from univariate logistic regression; aOR = adjusted odds ratio from multiple logistic regression including all variables presented in this table, but not all questionnaire variables (see Statistical analysis section); CI = Confidence interval;

a p-value from Chi-square test;

b p-value from the Wald’s test.

c None: farms that had never initiated the certification process. In process: Farms currently applying for or actively participating in the certification program. Certified: Farms that had fully achieved and maintained official certification status from the National Department of Livestock Development (DLD).

† Knowledge and attitude models include all farmers (N = 385). The combined preventive practice model is restricted to the predefined high-exposure subgroup only (farmers assisting animal births; N = 315).

### Awareness, knowledge, and attitude domains associated with preventive practices

Analysis of associations between farmers’ awareness, specific knowledge domains, and attitudes revealed their distinct contributions to preventive practices against occupational brucellosis (Fig 3A, 3B, 3C). Farmers who had heard of the scientific term “brucellosis” (aOR 1.94, 95% CI: 1.12-3.38) had significantly higher odds of satisfactory knowledge (Fig 3A). Those who had heard of local alerts about brucellosis in livestock were more likely to have prevention-focused attitude (overall combined positive attitude; aOR 4.61, 95% CI: 2.56-8.29). Knowledge of farm biosecurity practices significantly increased the odds of a prevention-focused attitude (aOR 3.35, 95% CI: 1.68-6.66). Knowledge of host range and zoonotic susceptibility (aOR 1.63, 95% CI: 0.88-3.02) and clinical manifestations in humans (aOR 1.59, 95% CI: 0.84-3.03) showed positive but not statistically significant associations with prevention-focused attitude (Fig 3B). For the adoption of preventive practice (Fig 3C), having a prevention-focused attitude regarding perceived occupational susceptibility to brucellosis (aOR 3.66, 95% CI: 1.26-10.63) and awareness of heard of the scientific term “brucellosis” (aOR 2.74, 95% CI: 1.00-7.46) showed borderline positive associations with favorable combined preventive practice outcomes.

**Fig 3:**
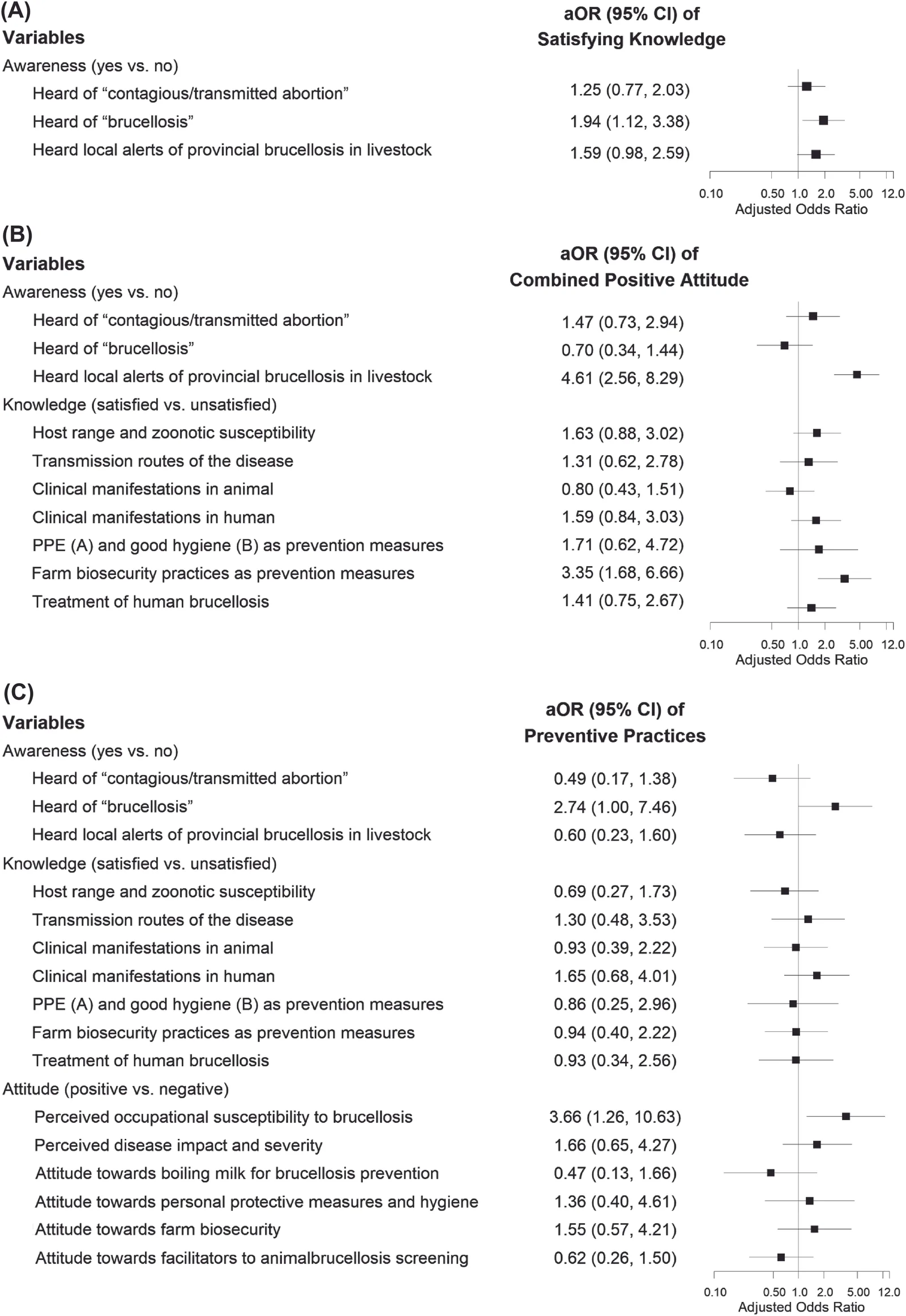
Forest plots illustrating the adjusted odds ratios (aORs) with 95% confidence intervals (CIs) from separate multivariate logistic regression models identifying the associations between. (A) satisfying knowledge and awareness domains regarding occupational brucellosis (n = 385); (B) combined positive attitude (prevention-focused attitude), adjusted for awareness and knowledge domains (n = 385); and (C) combined preventive practices, adjusted for awareness, knowledge, and prevention-focused attitude domains (n = 315) among ruminant farmers. Models were adjusted for all covariates shown in each panel. aOR: adjusted odds ratio; CI: confidence interval. Analysis and figure produced in RStudio version 2025.05.1 + 513 for Windows.

## Discussion

This study provides a comprehensive characterization of the determinants influencing occupational brucellosis prevention behaviors among ruminant farmers in Northeastern Thailand, utilizing a KAP framework. Our findings critically demonstrate that while general awareness of brucellosis exists, it often fails to translate into consistent protective behaviors. Instead, we identified specific knowledge of brucellosis transmission routes and targeted preventive attitudes as the strongest predictors of favorable practices.

### Farmers’ sociodemographic and structural determinants

In our study, small ruminant (goat, sheep) farmers demonstrated significantly stronger knowledge regarding brucellosis prevention compared to large ruminant (buffalo, cattle) farmers. This finding is consistent with occupational risk data, which identifies *Brucella melitensis*, commonly found in goats and sheep, as highly transmissible to humans worldwide [18,27–29]. In endemic areas, public health authorities often prioritize resources and veterinary campaigns for small ruminant brucellosis due to its zoonotic significance. These efforts, including intensive educational materials and disease management messaging specifically targeting sheep and goat farmers, likely explain the observed differences in knowledge and attitudes [18,30]. Mechanistically, small ruminant farming typically involves confined housing and frequently close contact, while large ruminant systems often use more extensive, less interactive management [30,31]. Prevention strategies must explicitly target large-ruminant farmers, who appear to receive less routine brucellosis messaging despite sustained occupational exposure.

Our study revealed that satisfactory knowledge of occupational brucellosis was significantly associated with farmers aged over 60 years, compared to younger groups. This is consistent with findings from prior research, showing older farmers in agricultural communities often possess greater disease-related knowledge in rural or endemic settings [27,32]. However, other reports indicate that age is not linked to higher knowledge levels regarding brucellosis prevention [30,33,34]. A plausible explanation for this age-related pattern is experiential learning. In brucellosis-endemic regions, cumulative occupational experience and community knowledge sharing often substitute for formal education, enabling older farmers to develop a practical understanding of brucellosis transmission routes and clinical signs through decades of practice [35,36]. Older farmers may also have had more frequent contact with livestock disease-control activities or veterinary extension programs over time, which were not explicitly captured in our questionnaire and could partly contribute to the observed age–knowledge pattern. This suggests that practical experience serves as a primary knowledge reservoir in this context. However, the wide confidence interval around the age effect indicates limited precision of this estimate, and the association should therefore be interpreted with caution. Therefore, prevention programs should not assume younger farmers are better informed but should instead formalize experience-based knowledge so it reaches those without historical exposure to brucellosis.

Despite the observed association between farmer’s age and knowledge, our findings revealed a notable disconnect between years of farming experience and adaptation of brucellosis preventive practices. While some studies have reported a positive relationship between experience and knowledge levels [30,37], our results indicate that, despite older farmers’ higher knowledge, accumulated farming experience did not consistently translate into safer practices. This observation echoes studies where traditional experience coexists with persistent risky behaviors [2,30]. Instead, brucellosis risk seems driven more by exposure intensity and type of animal contact than by years in farming [38]. These findings highlight the critical role of formal veterinary training and community outreach, which improve KAP more effectively than experience alone, and emphasize the need for tailored awareness programs for all farmers, regardless of experience [2,30].

Our study found that farmers with diversified income sources showed significantly better occupational brucellosis knowledge, while higher education was associated with more preventive attitudes toward the disease. This conceptual link is consistent with several indirect studies. Households with more diversified livelihoods and the means to invest in education are more likely to adopt modern farming practices and access formal animal and human health information [31,39]. In contrast, a KAP study among Thai goat farmers found that lower education was linked to poorer KAP on occupational brucellosis, with information mainly obtained informally from peers or family [19]. Together, these findings position education and livelihood diversification as key components of human capital, which, if not supported by adequate infrastructure, can limit farmers’ ability to translate knowledge and attitudes into effective preventive practices [31,40,41]. This underscores the need to prioritize tailored training and to establish easily accessible formal information channels for households with lower education and less diversified income to strengthen occupational brucellosis prevention and control.

Farm certification under GFM and GAP standards issued by the DLD in Thailand functions as a proxy for access to formal training, veterinary oversight, and standardized biosecurity, which are key to reducing brucellosis risk at both individual and farm levels [42]. In our study, certified farms reported significantly better preventive practices than non-certified farms. This structural effect likely reflects the individual-level mechanism seen in our K-A results, where better biosecurity knowledge was associated with higher odds of prevention-focused attitudes. This aligns with findings from Bangladesh, where farmers who received biosecurity training had markedly higher good-knowledge scores (25.8% vs 6.2%) and positive attitudes (up to 89.3%) [21]. Similarly, in Kenya, specific knowledge of brucellosis prevention methods was the key differentiator between safe and unsafe attitudes [28]. However, although knowledge helps shape positive attitudes, turning those attitudes into consistent practice usually requires more than awareness alone. In South Africa, for example, high general awareness did not guarantee positive attitudes when economic or structural barriers conflicted with recommended practices [35]. This implies that certification programs are effective not only because they deliver information, but because they create structural conditions, such as regular audits and strict quarantine procedures, enabling knowledge to be translated into routine practice [43]. Consequently, these programs generate a feedback loop that reinforces favorable behaviors, such as the use of protective gear and early disease detection, which are proven to lower occupational *Brucella* exposure among livestock farmers [33].

Despite its benefits, certification uptake in our study was low, with fewer than one-fifth of farms holding DLD certification. Prior research suggests that participation in certification schemes is often constrained by regulatory and economic barriers [44]. Moreover, sustained adherence to recommended practices requires ongoing veterinary support to reinforce disease awareness among farmers [43]. To maximize the contribution of farm certification on systemic brucellosis control within a One Health framework, future programs should focus on improving accessibility and providing targeted incentives, particularly for smallholder farms.

### Cognitive and attitudinal drivers

In our study, farmers who received local disease alerts and had a clearer understanding of farm‑level biosecurity tended to report more prevention‑focused attitudes. These attitudes, in turn, can contribute to favorable preventive behavior among farmers. However, our finding also revealed critical knowledge gaps concerning zoonotic host range (32.7%), clinical manifestations in humans (38.4%), and treatment (25.1%) of occupational brucellosis. Prior study in Thailand showed that 46% of goat farmers did not know the symptoms of human brucellosis, leading to a perception of zero risk [19]. Consistent findings from Saudi Arabia showed that lack of education was significantly associated with poor knowledge of human brucellosis transmission and symptoms [30]. These specific gaps undermine early health care‑seeking and community‑level control, ultimately perpetuating the brucellosis burden in the community.

While most brucellosis and zoonosis KAP studies report high proportions of positive attitudes (approximately 50–80%) even with suboptimal practices [21,45,46]. Only 1.6% of our surveyed farmers exhibited an overall positive preventive attitude toward occupational brucellosis, revealing a major attitudinal gap in our setting. This aligns with the PRECEDE-PROCEDE study of occupational brucellosis in Iran, which reported low attitude scores, especially regarding proactive prevention measures [47], which indicates that there was a critical bottleneck at the attitudinal level. In contrast, KAP study in Bangladesh showed that 86.3% of farmers reported a positive attitude toward zoonosis control despite poor hygiene practices [21]. Similar with KAP study in South Africa, which reported 91.6% farmers gained higher attitudinal concern as their perception of serious financial threat due to zoonotic disease burden [35]. This severe attitudinal gap, echoing findings, indicates a critical engagement barrier in our cohort, rather than merely a knowledge-to-practice translation challenge.

Regarding the adoption of preventive practice, overall knowledge was not significantly predictor. Instead, domain-specific knowledge, particularly on brucellosis transmission routes (77.8%), and a positive preventive attitude toward foodborne transmission (76.2%) were more strongly associated with comprehensive preventive practices at both personal and farm levels [27,45,48,49]. This suggests that general awareness is insufficient; precise, targeted risk education plus structural support is essential. Awareness of the term “brucellosis” aligned with satisfactory knowledge, while awareness of local alerts aligned with preventive attitudes, underscoring the key role of formal information channels in shaping risk perception and behavior change [28,41,49].

### Behavioral outcome and translation to practice

Despite the low overall adoption of comprehensive practices at the person and farm level (63/315, 20.0%), farmers frequently reported wearing boots during high-risk activities, while glove use was notable less common. This selective engagement, prioritizing perceived practicality over essential protection aligns with similar finding in Thailand, Bangladesh and Greece, where resistance to glove use is often linked to practical constrains or accessibility issues [19,21,31]. This suggests that enabling factors, such as ease of use and physical accessibility, may exert stronger influence on preventive behavior than knowledge or attitude alone [47].

Our findings identified transmission route knowledge (aOR 2.74) and positive attitudes toward occupational susceptibility (aOR 3.66) as the strongest independent associated factors of improved occupational brucellosis preventive behavior, with perceived severity showing a moderate association. These results support the Health Belief Model (HBM), which suggests perceived susceptibility as a primary driver of modifying behavior, consistent with a previous study on unsafe placenta disposal linked to specific knowledge gaps [41,50].

Notably, no surveyed farmers reported consuming raw milk or placental products, a significant contrast to nomadic pastoralists in Kenya (79.5%) and Ethiopia (68.3%) where raw milk consumption is common [28,49]. As unpasteurized dairy consumption substantially increases brucellosis seropositivity, its absence in our setting effectively neutralizes a major transmission vector [33]. Consequently, direct occupational contact during parturition assistance and birth product handling emerges as the predominant remaining exposure route, rendering the persistent low use of glove a critical vulnerability [33,45,51].

Importantly, adopting these protective behaviors provides immediate cross-protection against Q fever (coxiellosis) caused by *Coxiella burnetii*. Because *C. burnetii* is shed heavily in placental tissues and birth fluids, it poses a severe occupational threat via direct contact or aerosol inhalation during animal delivery. This overlapping risk is well-documented in Thailand, where concurrent seropositivity against both brucellosis and coxiellosis occurs within smallholder networks [10]. Most critically, recent Thai clinical data molecularly confirmed an actual human co-infection of *B. melitensis* and *C. burnetii* [52]. Since both pathogens share identical transmission pathways during parturition management, framing public health training around a dual-pathogen protection model could significantly increase smallholders’ perceived risk and boost routine glove and boot compliance.

To effectively address these behavioral gaps and enhance brucellosis prevention, targeted interventions are critical. The HBM explains why knowledge alone often proves insufficient for behavioral change, as perceived barriers including limited access to affordable PPE and competing economic priorities and broader structural constraints frequently supersede individual cognitive factors [53]. Therefore, we advocate adapting theory-driven strategies, similar to successful HBM-based interventions [50,54], to specifically tackle the attitudinal bottlenecks and structural barriers identified in Northeastern Thailand and similar agricultural contexts.

### Strength and limitation of the study

This study significantly addresses evidence gaps by examining occupational brucellosis prevention determinants among ruminant farmers in Northeastern Thailand, a region where epidemiological data on farmer KAP remain limited. Nevertheless, several limitations warrant acknowledgement. Firstly, the cross-sectional design inherently limits causal inference, making it difficult to determine whether increased knowledge drives certification and behavioral changes, or instead reflects prior engagement with veterinary and extension services. Secondly, the use of convenience sampling and voluntary participation may have introduced selection bias, potentially over-representing farmers with stronger connections to veterinary services and more favorable attitudes. The under-representation of small ruminant owners further limits generalizability, potentially inflating observed KAP disparities. Thirdly, although the achieved sample size met the original calculation based on glove use prevalence, it was not specifically powered for comparative subgroup analyses or multivariable logistic regression. Consequently, some subgroup estimates, particularly those stratified by age group, livestock ownership, farm certification status, and the predefined high-exposure subgroup for combined preventive practices, are based on small numbers. This limits statistical power, yields wide confidence intervals, and requires cautious interpretation. In addition, the exclusion of approximately 18% of respondents from the combined preventive practice outcome due to incomplete data on high-risk farm activities could have introduced selection bias. The low prevalence of certified farms and small ruminant ownership constrained the sensitivity and precision of subgroup analyses for these specific predictors. Fourthly, as all KAP measures were self-reported, there is a potential for social desirability bias and recall errors, which could affect the accuracy of estimates across all respondent groups. Additionally, as recruitment was conducted exclusively through the DLD‑registered farmer network, only farmers with Thai nationality were included. Therefore, unregistered farmers and migrant agricultural workers were not represented in this study. This potentially limits the generalizability of our findings to these populations, who likely possess distinct risk profiles and preventive practices. Finally, the quantitative questionnaire did not fully capture crucial enabling factors, such as economic constraints, market access, health system infrastructure, or broader social and cultural determinants of risk perception. Consequently, these findings should be interpreted as context-specific, non-causal evidence suitable for hypothesis generation and intervention design, rather than definitive population estimates or causal effects.

Despite these limitations, this study possesses several noteworthy strengths. It contributes to the literature by specifically focusing on occupational brucellosis among livestock farmers in a data-scarce region, revealing how structural factors interact with individual-level elements to shape preventive practices. A key methodological strength is the integration of visual aids within a standardized, structured questionnaire. This robust mechanism effectively minimized information bias and successfully navigated terminology barriers frequently encountered in agricultural contexts [24,35]. This approach underscores the potential for interactive educational tools to enhance accessibility for farmers and local stakeholders beyond traditional text-heavy dissemination.

### Implication of study

Our findings identify farm certification as a critical structural lever for enhancing occupational safety. Consequently, interventions should prioritize domain-specific training (e.g., on transmission, symptoms, and appropriate PPE use), specifically targeting non-certified and lower-education farmers. Such training would be most effectively delivered via formal channels, such as Village Health Volunteers (VHVs), rather than relying on informal networks. Structurally, we advocate for expanding certification access through mechanisms like smallholder subsidies or simplified procedures. Integrating these incentives into national One Health strategies will be crucial for aligning institutional biosafety frameworks with on-farm risk reduction efforts. To operationalize these interventions, Thailand’s twin networks of community volunteers offer an ideal grassroots infrastructure. Within the human health sector, VHVs excel in community health education and local syndromic surveillance [55]. Parallelly, the Department of Livestock Development relies on Livestock Volunteers (LVs) for village-level herd surveillance, animal disease reporting, and farmer training. Integrating both networks bridge the gap between human and veterinary sectors [56]. Jointly training VHVs to flag household occupational risks and LVs to enforce on-farm PPE compliance creates a practical, community-level One Health collaboration that effectively delivers targeted biosecurity education to lower-education smallholders.

### Recommendation

Future research should employ probability sampling and qualitative methods (e.g., in-depth interviews, focus groups) to better elucidate the socioeconomic barriers hindering adoption of preventive practices. Beyond descriptive KAP assessments, subsequent studies should integrate behavioral frameworks, such as the Health Belief Model or Theory of Planned Behavior, to comprehensively understand motivations and barriers. The scope should expand to include veterinary professionals, abattoir workers, and other occupational groups with zoonotic exposure, adopting a One Health perspective. Finally, economic evaluations of policy interventions, such as certification programs, are essential to validate the feasibility and sustainability of these occupational health initiatives.

## Conclusions

In conclusion, this study identifies critical determinants of occupational brucellosis prevention among ruminant farmers. Our findings underscore farm certification, higher education, and small ruminant ownership as key enablers of safer practices, while economic dependence solely on livestock farming presents a significant barrier. Crucially, despite exhibiting moderate knowledge levels, few farmers consistently demonstrated favorable preventive behaviors, highlighting persistent attitudinal and behavioral gaps. The robust influence of farm certification strongly affirms the efficacy of institutional oversight and structured training in fostering sustained biosecurity compliance. Therefore, these findings advocate for certification-linked interventions, integrated within a One Health framework, as a strategic priority to strengthen brucellosis control and significantly enhance occupational safety in livestock production.

## Methods

### Ethics statement

Ethics approval was obtained from the Ethical Review Committee for Human Research, Faculty of Public Health, Mahidol University (COA. No. MUPH 2024–086), prior to the data collection. All participation was voluntary and written informed consent obtained after both verbal and written explanations of the study. Farmers were informed of their right to refuse or withdraw at any point during interviews. To ensure confidentiality, no personal identifiers were recorded, and all data were stored anonymously. Results are presented in aggregate form without identifying or referring to any specific individuals.

### Study design, setting and population

This study employed a cross-sectional design to investigate the knowledge, attitudes, and practices (KAP) related to brucellosis control and prevention among livestock farmers in the lower northeastern region of Thailand (Fig 1) Three provinces including Nakhon Ratchasima, Surin, and Buriram, were strategically selected for their high livestock productivity and extensive farmer populations, based on data from the Department of Livestock Development (DLD) [1]. The reference population comprised ruminant livestock farmers (raising beef cattle, dairy cattle, buffalo, goats, or sheep) registered in the DLD database within the selected provinces. According to 2023 DLD data, the total population of ruminant livestock farmers in these three provinces was 351,979 individuals, reflecting a substantial agricultural base [22].

### Sample size and participant recruitment

The sample size was calculated using Cochran’s formula for a cross-sectional study, with a 95% confidence level and a 5% margin of error. An estimated proportion (p = 0.24) for preventive glove use was derived from a previous KAP study in Thailand [19]. This yielded an initial sample size of 281 participants. To account for potential non-response rates and incomplete data collection, an additional 10% was added, resulting in a final target sample size of 308 participants. The study population comprised ruminant livestock farmers registered in the DLD database in the three provinces. Participants were recruited using convenience sampling through coordination with provincial livestock officers and routine extension activities between 1 October 2024 and 31 July 2025. During these visits, eligible Thai farmers registered in the DLD database were invited to participate the study. Each interview was conducted with the main ruminant livestock farmer in the household, and each registered farm household contributed only one farmer to the study. No quotas were imposed for specific livestock species. Data were collected through face-to-face interviews conducted by trained research assistants. Ultimately, 385 farmers were included in the final analytical sample after data cleaning.

### Data collection tool

A structured questionnaire was developed and administered through face-to-face interviews, ensuring standardized questions for consistent responses across participants and facilitating systematic comparisons across provinces. This approach also helped achieve higher response rates and accommodate participants with varying literacy levels [23]. The questionnaire was systematically designed to comprehensively assess the three key constructs of the KAP framework; knowledge, attitude, and practices. The questionnaire comprised 113 questions organized into four distinct sections: [1] sociodemographic and farm characteristics (27 items) [2], self-reported practices related to brucellosis control and prevention (25 items) [3], attitudes about brucellosis risks, control measures and prevention (26 items), and [4] awareness and knowledge about brucellosis (35 items).

The questions were predetermined with structured response formats, primarily utilizing multiple-choice responses and Likert-type scales (see S1 File). To enhance data quality and response consistency, section-specific visual guides were developed as supplementary tools. These guides standardize response scales and facilitate participation across varying literacy levels (see S1 Fig) [24].

### Pretesting, data collection, and quality control

Pilot testing was conducted among twenty farms to assess question clarity, response formats, and field feasibility. Systematic feedback was used to revise items for legibility, coherence, and cultural appropriateness. Content validity was quantitatively assessed using the Index of Item-Objective Congruence (IOC). Three subject-matter experts (a medical professor, a senior public health veterinarian, and a livestock veterinary practitioner) independently rated each item using a standard IOC rating scale of +1/0/-1 scale. Items with IOC values ≥0.50 were considered acceptable, while those with values <0.50 required revision or removal. In this study, the final questionnaire demonstrated strong content validity, with IOC values of 0.80, 0.91, 0.86, and 0.91 for sections 1–4, respectively, yielding an overall IOC of 0.89. Internal consistency was evaluated using Cronbach’s alpha coefficient (α), yielding values of 0.84 for knowledge section, 0.80 for attitude section, 0.8 for practice section, and 0.82 for the overall questionnaire, considering good reliability. The final validated questionnaire required 20–30 minutes for completion minimizing participant burden.

### Statistical analysis

Farmers’ sociodemographic characteristics and farm-related variables were analyzed descriptively. Frequencies and percentages were used for categorical variables, whereas means and standard deviations were used for continuous variables. Ruminant species were categorized as large (beef cattle, dairy cattle, and buffalo) or small (goats and sheep) based on differences in husbandry practices, farm certification, disease risks, and economic importance relevant to brucellosis control.

Negative items were reverse-coded to ensure consistent scoring direction. Five-point Likert scales for attitude and practice variables were transformed into binary outcomes for logistic regression and clarity of interpretation. Higher scores in knowledge, attitude, and practice domains reflected greater knowledge, more positive attitudes, and better adherence to favorable practices.

Knowledge scores were classified as satisfying (≥70%) or unsatisfying (<70%). Attitudes were dichotomized by grouping “strongly agree” and “agree” as positive, with all other responses as negative. Practice variables were coded as “always” and “often” were coded as favorable, while “sometimes,” “rarely,” and “never” were considered as unfavorable. Within each attitude domain, a combined positive attitude or favorable practice required consistently positive responses across all key items (Table 1). The prevention‑focused attitude outcome was defined as the overall combined positive attitude toward occupational brucellosis, classified as positive only when all four attitude domains were favorable.

Farmers who reported performing animal birth assistance and parturition management within the past year were prespecified as a high-exposure subgroup for the analysis of person- and farm-level preventive practice during these tasks. Accordingly, farmers who did not report involvement in these high-risk activities were excluded from the combined preventive practice outcome analysis. Within this high-exposure subgroup, the overall combined preventive practice outcome represented comprehensive adherence to brucellosis preventive measures, requiring favorable responses for all key personal and farm-level practice items described in Table 1.

Associations between knowledge, attitudes, and preventive practices were examined using Chi-square or Fisher’s exact tests, as appropriate. Univariate logistic regression was used to estimate unadjusted associations (crude odds ratios; cORs) between each outcome and its predictors. Multivariate logistic regression was then performed separately for each outcome domain to estimate adjusted odds ratios (aORs). Analyses of satisfactory knowledge and prevention-focused attitude included all enrolled farmers, whereas the combined preventive practice outcome was analysed in a prespecified high-exposure subgroup of farmers who reported assisting animal births within the previous year, because birth-related practice items were only applicable to this subgroup. Covariates for each multivariable model were selected a priori based on theoretical plausibility and prior evidence regarding determinants of KAP outcomes in occupational zoonotic disease settings [25]. All statistical analyses were performed using R statistical software version 4.3.1 [26], with statistical significance set at p-value <0.05 and 95% confidence intervals (CIs) reported for all effect estimates.

## Supporting information

S1 Data Available data. (CSV)

S1 File KAP questionnaire on brucellosis in livestock farmers in Northeastern Thailand. (DOCX)

S1 Fig Visual guide to response scales for KAP questionnaire. (DOCX)

S1 Table Awareness and knowledge regarding occupational brucellosis among ruminant farmers (N = 385). (DOCX)

S2 Table Positive attitudes regarding occupational brucellosis among ruminant farmers (N = 385). (DOCX)

S3 Table Favorable preventive practices for occupational brucellosis prevention and control among ruminant farmers (N = 315). (DOCX)

S4 Table Awareness and satisfactory knowledge regarding favorable preventive practices for occupational brucellosis among ruminant farmers (N = 315). (DOCX)

S5 Table Positive attitude regarding favorable preventive practices for occupational brucellosis among ruminant farmers (N = 315). (DOCX)
