# Supplementary material for: Occupational brucellosis among livestock farmers in Northeastern Thailand: A cross-sectional study of knowledge, attitudes, and practices from a One Health perspective
Source: PLoS Negl Trop Dis. 2026 Oct 27;20(10):e0014788. doi: 10.1371/journal.pntd.0014788 (PMC13712670; doi:10.1371/journal.pntd.0014788)
Supplement: Supplementary file 1 [file pntd.0014788.s002.docx]

**S1 File.**

**KAP on Brucellosis in Livestock Farmers in Northeastern Thailand**

**^a^** possibility to choose more than one option

**Section 1: Demographics and Farm Characteristics**

1. Gender: Male ( ) Female ( )
2. Age: _______ years old
3. Marital status: Single ( ) Married ( ) Divorced ( ) Widowed ( ) Other ( )
4. Education level: No formal education ( ) Elementary school ( ) Junior high school ( ) High school ( ) High vocational school ( ) Bachelor's degree ( ) Master's degree ( ) Doctoral degree or above ( )
5. Religion: Buddhist ( ) Christian ( ) Muslim ( ) No religion ( ) Other ( )
6. What types of ruminant animals do you raise?^a^ Beef cattle ( ) Dairy cattle ( ) Buffalo ( ) Goats ( ) Sheep ( )
7. Is livestock farming your only source of income? Yes ( ) No ( )
8. Do you live on your farm?

Yes ( ) (Within a distance of not more than 1 kilometer, Between 1-5 kilometers, More than 5-10 kilometers)

No ( )

9. How many people live in your household (including yourself)? _______ people
10. How many people help you work on your farm (including yourself)? _______ people
11. How many households help you work on your farm (including your household)? _______ households
12. How many outside workers who are not a family member help you work on your farm? _______ people
13. How long have you been working in livestock farming? _______ years
14. How long has your farm been in operation? _______ years
15. How did you acquire your farm? Own farm( ) Inherited ( ) Purchased ( ) Other ( )
16. How do you breed your animals?^a^

Mating within the farm ( )	Natural mating ( ) Artificial insemination ( )

Mating outside the farm ( )	Natural mating ( ) Artificial insemination ( )

17. How many animals do you currently have on your farm (on the interview day)?

Beef cattle ( ) _______ amount

Dairy cattle ( ) _______ amount

Buffalo ( ) _______ amount

Goats ( ) _______ amount

Sheep ( ) _______ amount

Other ( ) _______ amount

18. The question is only for dairy cow famer. if you are not a dairy cow farmer, skip to question 19.

How many of your dairy cows are currently lactating?

Number of gestating cows _______ cows

Number of lactating cows_______ cows

19. Is your farm certified by the Department of Livestock Development?

(GFM : Good Farming management, GA: Good Agricultural Practice)

Beef cattle ( ) Yes ( ) No ( )

GFM ( )	( ) Farm standard has been certified in year ....

( ) In the process of applying for standard certification

( ) In the process of preparing the farm's readiness

( ) No plans to apply for standard certification

GAP ( )	( ) Farm standard has been certified in year ....

( ) In the process of applying for standard certification

( ) In the process of preparing the farm's readiness

( ) No plans to apply for standard certification

Free Brucellosis farm ( )	( ) Farm standard has been certified in year ....

( ) In the process of applying for standard certification

( ) In the process of preparing the farm's readiness

( ) No plans to apply for standard certification

Dairy cattle ( )

GFM ( )	( ) Farm standard has been certified in year ....

( ) In the process of applying for standard certification

( ) In the process of preparing the farm's readiness

( ) No plans to apply for standard certification

GAP ( )	( ) Farm standard has been certified in year ....

( ) In the process of applying for standard certification

( ) In the process of preparing the farm's readiness

( ) No plans to apply for standard certification

Free Brucellosis farm ( )	( ) Farm standard has been certified in year ....

( ) In the process of applying for standard certification

( ) In the process of preparing the farm's readiness

( ) No plans to apply for standard certification

Buffalo ( )

GFM ( )	( ) Farm standard has been certified in year ....

( ) In the process of applying for standard certification

( ) In the process of preparing the farm's readiness

( ) No plans to apply for standard certification

GAP ( )	( ) Farm standard has been certified in year ....

( ) In the process of applying for standard certification

( ) In the process of preparing the farm's readiness

( ) No plans to apply for standard certification

Free Brucellosis farm ( )	( ) Farm standard has been certified in year ....

( ) In the process of applying for standard certification

( ) In the process of preparing the farm's readiness

( ) No plans to apply for standard certification

Goats ( )

GFM ( )	( ) Farm standard has been certified in year ....

( ) In the process of applying for standard certification

( ) In the process of preparing the farm's readiness

( ) No plans to apply for standard certification

GAP ( )	( ) Farm standard has been certified in year ....

( ) In the process of applying for standard certification

( ) In the process of preparing the farm's readiness

( ) No plans to apply for standard certification

Free Brucellosis farm ( )	( ) Farm standard has been certified in year ....

( ) In the process of applying for standard certification

( ) In the process of preparing the farm's readiness

( ) No plans to apply for standard certification

Sheep ( )

GFM ( )	( ) Farm standard has been certified in year ....

( ) In the process of applying for standard certification

( ) In the process of preparing the farm's readiness

( ) No plans to apply for standard certification

GAP ( )	( ) Farm standard has been certified in year ....

( ) In the process of applying for standard certification

( ) In the process of preparing the farm's readiness

( ) No plans to apply for standard certification

Free Brucellosis farm ( )	( ) Farm standard has been certified in year ....

( ) In the process of applying for standard certification

( ) In the process of preparing the farm's readiness

( ) No plans to apply for standard certification

20. How would you describe the location of your farm and residence?

( ) Your farm located in a residential area.

( ) Your farm separately located from a residential area without a fence.

( ) Your farm separately located from a residential area with a fence.

21. Do you raise any other animals on your farm?

( ) Pigs ( ) Dogs/cats ( ) Birds ( ) Other

22. Do other animals enter and exit your farm? Yes ( ) No ( )
23. Do you have a quarantine area for new animals? Yes ( ) No ( )
24. Has your farm ever had a case of abortion? Yes ( ) No ( )
25. Do you have a pasture area in your farm? Yes ( ) No ( )
26. Where do you get your hay from?

( ) Nearby farm ( ) Other province ( ) Public pasture

27. Do you have preventive and management measures in place for your farm in the event of a brucellosis outbreak in the surrounding area?

( )Yes ( ) No

**Section 2: Behaviors or Practices Related to Brucellosis Control and Prevention**

**Instructions:** Please mark ✓ in the space that corresponds to your practices during the past 1 month. Choose only one answer using the following scale:

- **Always:** Means the activity is performed every time.
- **Often:** Means the activity is performed frequently (5 to 6 times per week).
- **Sometimes:** Means the activity is performed occasionally (3 to 4 times per week).
- **Rarely:** Means the activity is performed infrequently (1 to 2 times per week).
- **Never:** Means the activity is not performed at all.

1. Do you wear gloves when assisting with animal births, with them readily available in the birthing area?
2. Do you wear boots when assisting with animal births, with them readily available in the birthing area?
3. Do you wash your hands before and after milking animals, with hand washing facilities available within the work area, such as a faucet and soap?

Note: If there is no milking on the farm, skip to the next question.

4. Do you clean the birthing area after moving the animal, with the cleaning equipment and disinfectant readily available in the birthing area?
5. Do you wear boots when working on your farm, with them readily available in the working area?
6. Do you regularly check the health of your animals?
7. Do you separate sick animals from the herd if you find a sick animal?
8. Do you contact a veterinarian if you find a sick animal?
9. Do you clean your body after working on your farm?
10. Do you quarantine new animals before introducing them to the herd?
11. Do you consume milk from your farm?

Note: If there is no milk on the farm, skip to the next question.

12. Do you give milk from your farm to other animals on your farm such as dogs and cats?
13. Do you boil raw milk before consuming it?

Note: If there is no milk on the farm, skip to the next question.

14. Do you consume animal placenta from your farm?

Note: If there is no animal placenta on the farm, skip to the next question.

15. Do you cook animal placenta before consuming it?

Note: If there is no animal placenta on the farm, skip to the next question.

16. Do you consume raw milk or animal placenta from other farms or sources?
17. Do you boil raw milk or cook animal placenta from other farms or sources before consuming it?
18. Do you keep records of sick animals on your farm?
19. Do you keep records of your animals for at least 3 years?
20. Do you clean and disinfect your animal pens?
21. Do you disinfect vehicles that enter your farm?
22. Do you have a policy to prevent unauthorized people from entering your farm?
23. Do you have a measure to prevent other animals such as birds or rat from entering your farm and pens?
24. Do you test new animals for brucellosis before introducing them to the herd?
25. Do you vaccinate your animals against brucellosis at least once a year?

**Section 3: Knowledge about Brucellosis**

1. Have you ever heard the term “Rok Tang Tid Tor” (literally “transmissible abortion”) before? Yes ( ) No ( )
2. Have you heard the specific term “brucellosis”? Yes ( ) No ( )
3. Is brucellosis caused by a virus? Yes ( ) No ( )
4. Dairy cows can get brucellosis? Yes ( ) No ( )
5. Buffalo cannot get brucellosis? Yes ( ) No ( )
6. Goats, and sheep are not susceptible to brucellosis. Yes ( ) No ( )
7. Animals like pigs, dogs and cats can get brucellosis. Yes ( ) No ( )
8. Brucellosis a zoonotic disease. Therefore, human can get brucellosis. Yes ( ) No ( )
9. Consuming raw milk can cause brucellosis. Yes ( ) No ( )
10. Contact with newborn animals, placentas, or body fluids of infected animals such as blood or secretion can cause brucellosis. Yes ( ) No ( )
11. Consuming newborn animals or raw placentas can cause brucellosis. Yes ( ) No ( )
12. Female animals infected with brucellosis have reduced milk production. Yes ( ) No ( )
13. Brucellosis can cause animals to abort their fetuses. Yes ( ) No ( )
14. Brucellosis can cause animals to have swollen testicles. Yes ( ) No ( )
15. Brucellosis can cause infertility in animals. Yes ( ) No ( )
16. Brucellosis can cause mastitis in animals. Yes ( ) No ( )
17. Animals infected with brucellosis cannot be cured. Yes ( ) No ( )
18. Absence of abortion in an animal guarantee that the animal does not have brucellosis. Yes ( ) No ( )
19. Brucellosis can cause orchitis in humans? Yes ( ) No ( )
20. Brucellosis can cause fever for 1-2 months in humans? Yes ( ) No ( )
21. Brucellosis can cause joint pain in humans? Yes ( ) No ( )
22. Brucellosis can cause night sweats in humans? Yes ( ) No ( )
23. Brucellosis can cause flu-like symptoms such as fever or coughing in humans? Yes ( ) No ( )
24. Brucellosis in humans can be cured. Yes ( ) No ( )
25. Wearing personal protective equipment such as boots and gloves can reduce the risk of brucellosis. Yes ( ) No ( )
26. New animals should be tested and quarantined before being introduced to the herd. Yes ( ) No ( )
27. Cleaning your body after contact with animals can reduce the risk of brucellosis. Yes ( ) No ( )
28. Disinfectants can kill the agent that cause brucellosis? Yes ( ) No ( )
29. There is a brucellosis vaccine for animal. Yes ( ) No ( )
30. Animal brucellosis can be prevented by vaccination. Yes ( ) No ( )
31. Brucellosis in humans does not require an extended treatment period. Yes ( ) No ( )
32. To receive farm standard certification from the Department of Livestock, the farm must be free from brucellosis. Yes ( ) No ( )
33. Farms certified by the Department of Livestock Development must be tested for brucellosis regularly, at least once a year. Yes ( ) No ( )
34. If there are sick animals suspected of brucellosis infection, it is not necessary to separate those animals from the herd. Yes ( ) No ( )
35. If there are dead animals suspected of brucellosis infection, it is necessary to dispose of the carcasses properly. Yes ( ) No ( )

**Section 4: Attitudes about Brucellosis (26 Questions)**

**Instructions:** Please mark ✓ in the space that corresponds to your opinion during the past 1 month. Choose only one answer using the following scale:

- **Strongly Agree:** Means the statement most closely aligns with your opinion.
- **Agree:** Means the statement closely aligns with your opinion.
- **Moderately Agree:** Means the statement moderately aligns with your opinion.
- **Slightly Agree:** Means the statement slightly aligns with your opinion.
- **Disagree:** Means the statement most strongly does not align with your opinion.

**Questions:**

1. How likely do you think it is that brucellosis is currently present in your herd?
2. How likely do you think it is that brucellosis is present in other animals on your farm?
3. Do you think you have a possibility of contracting brucellosis?
4. Do you think that farmers who raise cattle, buffalo, goats, and sheep are at a higher risk of contracting brucellosis than other occupations?
5. Do you think that farmers who raise other animals, such as pigs, are at a higher risk of contracting brucellosis than other occupations?
6. Do you think that people in other professions who are not involved with animals have the same risk of contracting brucellosis as those who work with animals?
7. Do you think your family members are at risk of contracting brucellosis?
8. Do you think families with pets are at a higher risk of contracting brucellosis than families without pets?
9. Do you think brucellosis has been detected in cattle, buffalo, goats, and sheep in your province?
10. Do you think it is unnecessary to boil milk before consumption?
11. Do you think new animals purchased or brought into the farm have a chance of having brucellosis?
12. Do you think it is a waste of labor to test animals on the farm for brucellosis?
13. Do you think it is a waste of time to screen animals on the farm for brucellosis?
14. Do you think it is a waste of money to screen animals on the farm for brucellosis?
15. Do you think it is a waste of labor to vaccinate against brucellosis?
16. Do you think it is a waste of time to vaccinate against brucellosis?
17. Do you think it is a waste of money to vaccinate against brucellosis?
18. Would you be willing to cooperate if there were free brucellosis testing or vaccination services?
19. Do you think it is necessary to notify a veterinarian or Department of Livestock officers if your farm animals are sick and suspected of having brucellosis?
20. Do you think it is troublesome to wash your hands after contacting animals?
21. Do you think it is unnecessary to use personal protective equipment such as boots and gloves when working?
22. Do you think it is unnecessary to use disinfectant on the farm?
23. Is brucellosis a disease that needs to be prioritized for control and prevention?
24. Do you think that new animals brought onto the farm can be purchased from any farm without needing to know their history?
25. Does brucellosis have an impact on the economy and health of both humans and animals?
26. Do you think you would like to know more about brucellosis?
