# Supplementary material for: Occupational brucellosis among livestock farmers in Northeastern Thailand: A cross-sectional study of knowledge, attitudes, and practices from a One Health perspective
Source: PLoS Negl Trop Dis. 2026 Oct 27;20(10):e0014788. doi: 10.1371/journal.pntd.0014788 (PMC13712670; doi:10.1371/journal.pntd.0014788)
Supplement: Supplementary file 2 [file pntd.0014788.s003.docx]

**S1 Fig.** Response scale visual guide for KAP questionnaire

**(1) Frequency of practice**

| **Always**  **10 out of 10 times** | **Often**  **7-9 out of 10 times** | **Sometimes**  **4-6 out of 10 times** | **Rarely**  **1-3 out of 10 times** | **Never** |
| --- | --- | --- | --- | --- |

**(2) Level of agreement**

**(5)	(4)	(3)	(2)	(1)**

**Strongly agree	Agree	Neutral	Disagree	Strongly disagree**

**(3) Knowledge:**

**No**

**Yes**
