# Supplementary material for: Occupational brucellosis among livestock farmers in Northeastern Thailand: A cross-sectional study of knowledge, attitudes, and practices from a One Health perspective
Source: PLoS Negl Trop Dis. 2026 Oct 27;20(10):e0014788. doi: 10.1371/journal.pntd.0014788 (PMC13712670; doi:10.1371/journal.pntd.0014788)
Supplement: Supplementary file 3 [file pntd.0014788.s004.docx]

**S1 Table**. Awareness and knowledge regarding occupational brucellosis among ruminant farmers (N=385)

| **Knowledge variables** | **Total**  **(N=385)** |
| --- | --- |
|  | **n (%)** |
| **Awareness of the disease** |  |
| *Awareness by brucellosis recognition* |  |
| Heard of “contagious/transmitted abortion” (local term), yes | 241 (62.6) |
| Heard of “brucellosis” (standard term), yes | 115 (29.9) |
| *Awareness by epidemiological risk of animal brucellosis* |  |
| Heard local alerts of provincial brucellosis in livestock, yes | 111 (28.8) |
| Overall awareness level, high | 71 (18.4) |
| **Satisfactory knowledge of brucellosis** |  |
| *Host range and zoonotic susceptibility* |  |
| Correctly identified susceptible hosts (e.g., cattle, goats) and animal-to-human transmission risk | 122 (31.7) |
| *Transmission routes of the disease* |  |
| Correctly identified the transmission risks of brucellosis from direct contact and consumption of animal’s raw product (milk, meat, placenta) | 257 (66.8) |
| *Clinical manifestations in animal* |  |
| Correctly identified animal clinical signs (e.g., abortion, orchitis, infertility) | 180 (46.8) |
| *Clinical manifestations in human* |  |
| Correctly identified common human symptoms (e.g., prolonged fever, night sweating, joint pain, orchitis) | 153 (39.7) |
| *Prevention and control* |  |
| Correctly identified using personal protective equipment (PPE) as a prevention measure | 363 (94.3) |
| Correctly identified hygiene (hand washing) and disinfection as prevention measures | 335 (87.0) |
| Correctly identified the key farm biosecurity measures (new animal quarantine, sick animal isolation, and carcass disposal) as prevention practices | 214 (55.6) |
| *Treatment of human brucellosis* |  |
| Correctly identified facts about human brucellosis treatment (curable, long duration) | 96 (24.9) |
| Overall knowledge level, satisfied^b^ | 252 (65.5) |

^a^ p-value by Chi-square test

^b^ Satisfying knowledge level is defined as a total correct response score of at least 18 out of 26 items (corresponding to at least 70%) on the knowledge assessment.
