# Supplementary material for: Occupational brucellosis among livestock farmers in Northeastern Thailand: A cross-sectional study of knowledge, attitudes, and practices from a One Health perspective
Source: PLoS Negl Trop Dis. 2026 Oct 27;20(10):e0014788. doi: 10.1371/journal.pntd.0014788 (PMC13712670; doi:10.1371/journal.pntd.0014788)
Supplement: Supplementary file 4 [file pntd.0014788.s005.docx]

**S2 Table.** Positive attitude regarding occupational brucellosis among ruminant farmers (N=385)

| **Attitude variables** | **Total**  **(N=385)** |
| --- | --- |
|  | **n (%)** |
| **AA: Perceived occupational susceptibility to brucellosis** |  |
| *Zoonotic susceptibility* |  |
| (a1.2) Ruminant farmers have a higher risk of contracting brucellosis than people in other occupations. | 141 (36.6) |
| (a1.3) Other livestock farmers, such as those raising pigs, have a higher risk of contracting brucellosis than people in other occupations. | 139 (36.1) |
| (a1.4) People in non-animal-related professions do not have the same risk level of contracting brucellosis as those who work directly with animals. | 252 (65.5) |
| Overall perceived susceptibility attitude, positive^b^ | 36 (9.4) |
| **AB: Perceived disease impact and severity** |  |
| *Disease impact and severity* |  |
| Brucellosis has an impact on the economy and health of both humans and animals. | 281 (73.0) |
| Brucellosis is a disease that needs to be prioritized for control and prevention. | 291 (75.6) |
| Overall perceived disease impact and severity, positive^b^ | 249 (64.7) |
| **AC: Attitude toward prevention and control** |  |
| *Recognition of risky transmission route* |  |
| It is necessary to boil milk before consumption to reduce the risk of brucellosis. | 277 (71.9) |
| *Personal protective measures and hygiene* |  |
| Washing hands after contacting animals is not troublesome. | 283 (73.5) |
| Using personal protective equipment, such as boots and gloves, is necessary when working with farm animals. | 284 (73.8) |
| Overall personal prevention attitude, positive^b^ | 204 (53.0) |
| *Farm biosecurity* |  |
| Using disinfectant on the farm is necessary. | 251 (65.2) |
| New animals purchased or brought into the farm may carry brucellosis and potentially transmit it to the existing herd. | 175 (45.5) |
| New animals brought onto the farm should not be purchased from any farm without knowing their history. | 244 (63.4) |
| It is necessary to notify a veterinarian or Department of Livestock officers if farm animals are sick and suspected of having brucellosis. | 316 (82.1) |
| Overall farm-level prevention attitude, positive^b^ | 90 (23.4) |
| Overall combined attitude toward prevention of brucellosis at personal and farm-level, positive^b^ | 67 (17.4) |
| **AD: Facilitators to animal brucellosis screening** |  |
| *Disease screening* |  |
| Testing animals on the farm for brucellosis is not a waste of labor. | 216 (56.1) |
| Screening animals on the farm for brucellosis is not a waste of time. | 213 (55.3) |
| Screening animals on the farm for brucellosis is not a waste of money. | 203 (52.7) |
| Farmers are willing to cooperate if free brucellosis testing services are provided. (Strategy to reduce barrier) | 328 (85.2) |
| Overall facilitators attitude, positive^b^ | 154 (40.0) |
| **Overall combined attitude toward occupational brucellosis, positive^b^** | **5 (1.3)** |

^b^ Positive attitude was defined as the combination of participants who responded "strongly agree" or "agree" to each statement.
