# Supplementary material for: Occupational brucellosis among livestock farmers in Northeastern Thailand: A cross-sectional study of knowledge, attitudes, and practices from a One Health perspective
Source: PLoS Negl Trop Dis. 2026 Oct 27;20(10):e0014788. doi: 10.1371/journal.pntd.0014788 (PMC13712670; doi:10.1371/journal.pntd.0014788)
Supplement: Supplementary file 5 [file pntd.0014788.s006.docx]

**S3 Table.** Favorable preventive practices for occupational brucellosis prevention and control among ruminant farmers (N=315)

| **Practice variables** | **Total** |
| --- | --- |
|  | **n (%)** |
| **Personal protective equipment (PPE) and hygiene practices** |  |
| *Personal level prevention during animal birth assistance management* |  |
| No, this activity was not reported. | 70 (18.2) |
| Yes, this activity was reported. | 315 (81.8) |
| Wearing gloves is adopted when assisting with animal births. | 122 (38.7) |
| Wearing boots is practiced when assisting with animal births. | 194 (61.6) |
| **Overall personal level prevention during birth assistance, favorable^a^** | **102 (32.4)** |
| *Personal level prevention in daily farm operations and practicing hygiene* |  |
| Wearing boots is practiced while working on the farm. | 276 (71.7) |
| Cleaning the body after farm work | 321 (83.4) |
| **Overall personal preventive practice and hygiene during daily farm activity, favorable^a^** | **255 (66.2)** |
| Overall combined preventive practice (only among those reporting all farm activities including birth assistance and hygiene), favorable^a^ | 80 (23.1) |
| **Farm biosecurity** |  |
| *Sanitation and disinfection practices* |  |
| Cleans and disinfects animal pens on the farm. | 235 (61.0) |
| Disinfects vehicles that enter the farm. | 102 (26.5) |
| Overall farm-level preventive practice, favorable^a^ | 89 (23.1) |
| **Overall combined preventive practice^b^, favorable^a^** | **63/315 (20.0)** |

^a^ Favorable practice was defined as grouping responses of “very likely” (7–8 out of 10 times, 70–80%) and “most likely” (9–10 out of 10 times, 90–100%) to each practice.

^b^ Denominator for overall preventive practice (n=315) was less than the total sample size (n=385) because only farmers with complete data on all personal and farm-level preventive practice items were included; records with missing data on any items were excluded from this analysis.
