# Supplementary material for: Occupational brucellosis among livestock farmers in Northeastern Thailand: A cross-sectional study of knowledge, attitudes, and practices from a One Health perspective
Source: PLoS Negl Trop Dis. 2026 Oct 27;20(10):e0014788. doi: 10.1371/journal.pntd.0014788 (PMC13712670; doi:10.1371/journal.pntd.0014788)
Supplement: Supplementary file 6 [file pntd.0014788.s007.docx]

**S4 Table.** Awareness and satisfactory knowledge regarding favorable preventive practice for occupational brucellosis among ruminant farmers (N=315)

| **Awareness and knowledge variables** | **Total** | **Person-level preventive practices** | | | | | | **Farm-level preventive practice** | | | **Both person and farm level** | | |
| --- | --- | --- | --- | --- | --- | --- | --- | --- | --- | --- | --- | --- | --- |
|  |  | **During animal birth assistance ^1^** | | | **During daily farm activities ^2^** | | | **Farm biosecurity adherence** | | | **Overall combined all domains of preventive practices** | | |
|  |  | **Unfavorable** | **Favorable** |  | **Unfavorable** | **Favorable** |  | **Unfavorable** | **Favorable** |  | **Unfavorable** | **Favorable** |  |
|  | **(N=315)** | **(n=213)** | **(n=102)** |  | **(n=99)** | **(n=216)** |  | **(n=147)** | **(n=168)** |  | **(n=252)** | **(n=63)** |  |
|  | **n (%)** | **n (%)** | **n (%)** | **p-value** | **n (%)** | **n (%)** | **p-value** | **n (%)** | **n (%)** | **p-value** | **n (%)** | **n (%)** | **p-value** |
| **Awareness of the disease** |  |  |  |  |  |  |  |  |  |  |  |  |  |
| *Awareness by brucellosis recognition* | 87 (27.6) | 53 (24.9) | 34 (33.3) | 0.151 | 23 (23.2) | 64 (29.6) | 0.297 | 32 (21.8) | 55 (32.7) | 0.041 | 64 (25.4) | 23 (36.5) | 0.108 |
| Heard of “contagious/transmitted abortion” (local term), yes | 195 (61.9) | 134 (62.9) | 61 (59.8) | 0.684 | 60 (60.6) | 135 (62.5) | 0.844 | 87 (59.2) | 108 (64.3) | 0.416 | 152 (60.3) | 43 (68.3) | 0.310 |
| Heard of “brucellosis” (standard term), yes | 90 (28.6) | 53 (24.9) | 37 (36.3) | 0.050 | 23 (23.2) | 67 (31.0) | 0.199 | 33 (22.4) | 57 (33.9) | 0.034 | 65 (25.8) | 25 (39.7) | 0.043 |
| *Awareness by epidemiological risk of animal brucellosis* |  |  |  |  |  |  |  |  |  |  |  |  |  |
| Heard local alerts of provincial brucellosis in livestock, yes | 97 (30.8) | 65 (30.5) | 32 (31.4) | 0.981 | 28 (28.3) | 69 (31.9) | 0.602 | 43 (29.3) | 54 (32.1) | 0.666 | 80 (31.7) | 17 (27.0) | 0.562 |
| Overall combined awareness level, high | 61 (19.4) | 43 (20.2) | 18 (17.6) | 0.703 | 18 (18.2) | 43 (19.9) | 0.837 | 27 (18.4) | 34 (20.2) | 0.782 | 49 (19.4) | 12 (19.0) | >0.999 |
| **Satisfactory knowledge of brucellosis** |  |  |  |  |  |  |  |  |  |  |  |  |  |
| *Host range and zoonotic susceptibility* |  |  |  |  |  |  |  |  |  |  |  |  |  |
| Correctly identified susceptible hosts (e.g., cattle, goats) and animal-to-human transmission risk | 103 (32.7) | 69 (32.4) | 34 (33.3) | 0.970 | 36 (36.4) | 67 (31.0) | 0.418 | 45 (30.6) | 58 (34.5) | 0.537 | 80 (31.7) | 23 (36.5) | 0.568 |
| *Transmission routes of the disease* |  |  |  |  |  |  |  |  |  |  |  |  |  |
| Correctly identified the transmission risks of brucellosis from direct contact and consumption of animal’s raw product (milk, meat, placenta) | 206 (65.4) | 135 (63.4) | 71 (69.6) | 0.337 | 62 (62.6) | 144 (66.7) | 0.567 | 87 (59.2) | 119 (70.8) | 0.040 | 157 (62.3) | 49 (77.8) | 0.031 |
| *Clinical manifestations in animal* |  |  |  |  |  |  |  |  |  |  |  |  |  |
| Correctly identified animal clinical signs (e.g., abortion, orchitis, infertility) | 136 (43.2) | 88 (41.3) | 48 (47.1) | 0.400 | 39 (39.4) | 97 (44.9) | 0.427 | 60 (40.8) | 76 (45.2) | 0.499 | 107 (42.5) | 29 (46.0) | 0.712 |
| *Clinical manifestations in human* |  |  |  |  |  |  |  |  |  |  |  |  |  |
| Correctly identified common human symptoms (e.g., prolonged fever, night sweating, joint pain, orchitis) | 121 (38.4) | 79 (37.1) | 42 (41.2) | 0.566 | 42 (42.4) | 79 (36.6) | 0.386 | 52 (35.4) | 69 (41.1) | 0.357 | 94 (37.3) | 27 (42.9) | 0.505 |
| *Prevention and control* |  |  |  |  |  |  |  |  |  |  |  |  |  |
| Correctly identified using personal protective equipment (PPE) as a prevention measure | 299 (94.9) | 202 (94.8) | 97 (95.1) | >0.999 | 89 (89.9) | 210 (97.2) | 0.013 | 137 (93.2) | 162 (96.4) | 0.296 | 237 (94.0) | 62 (98.4) | 0.210F |
| Correctly identified hygiene (hand washing) as prevention measures | 271 (86.0) | 184 (86.4) | 87 (85.3) | 0.930 | 74 (74.7) | 197 (91.2) | < 0.001 | 125 (85.0) | 146 (86.9) | 0.753 | 217 (86.1) | 54 (85.7) | >0.999 |
| Correctly identified the key farm biosecurity measures (new animal quarantine, sick animal isolation, and carcass disposal) as prevention practices | 179 (56.8) | 122 (57.3) | 57 (55.9) | 0.911 | 59 (59.6) | 120 (55.6) | 0.583 | 84 (57.1) | 95 (56.5) | >0.999 | 143 (56.7) | 36 (57.1) | >0.999 |
| Overall combined knowledge in prevention and control, satisfied | 159 (50.5) | 107 (50.2) | 52 (51.0) | 0.997 | 47 (47.5) | 112 (51.9) | 0.549 | 76 (51.7) | 83 (49.4) | 0.769 | 125 (49.6) | 34 (54.0) | 0.632 |
| *Treatment of human brucellosis* |  |  |  |  |  |  |  |  |  |  |  |  |  |
| Correctly identified facts about human brucellosis treatment (curable, long duration) | 79 (25.1) | 56 (26.3) | 23 (22.5) | 0.563 | 28 (28.3) | 51 (23.6) | 0.454 | 35 (23.8) | 44 (26.2) | 0.722 | 64 (25.4) | 15 (23.8) | 0.922 |
| **Overall knowledge level, satisfying^b^** | **202 (64.1)** | **133 (62.4)** | **69 (67.6)** | **0.438** | **61 (61.6)** | **141 (65.3)** | **0.615** | **92 (62.6)** | **110 (65.5)** | **0.677** | **158 (62.7)** | **44 (69.8)** | **0.363** |

^1^ Only farmers reporting animal birth assistance activities were included. Favorable personal preventive practice was defined as simultaneous adoption of both glove use and boot use during birth assistance.

^2^ Favorable status reflected farmers who reported both regular use of boots during farm work and personal hygiene after farm activity; included all respondents (n=385) engaged in daily farm operations.

^3^ Favorable status for overall combined preventive practices was defined as farmers meeting criteria for all of: favorable personal preventive practice during animal birth assistance, daily farm work, and farm biosecurity adherence domains.

^a^ p-values by Chi-square test unless otherwise indicated.

^F^p-values by Fisher’s exact test.

^b^Satisfying knowledge level is defined as a total correct response score of at least 18 out of 26 items (corresponding to at least 70%) on the knowledge assessment.
