# Supplementary material for: Occupational brucellosis among livestock farmers in Northeastern Thailand: A cross-sectional study of knowledge, attitudes, and practices from a One Health perspective
Source: PLoS Negl Trop Dis. 2026 Oct 27;20(10):e0014788. doi: 10.1371/journal.pntd.0014788 (PMC13712670; doi:10.1371/journal.pntd.0014788)
Supplement: Supplementary file 7 [file pntd.0014788.s008.docx]

**S5 Table.** Positive attitude regarding favorable preventive practice for occupational brucellosis among ruminant farmers (N=315)

| **Attitude variables** | **Total** | | **Person-level preventive practices** | | | | | | **Farm-level preventive practice** | | | **Both persona and farm level** | | |
| --- | --- | --- | --- | --- | --- | --- | --- | --- | --- | --- | --- | --- | --- | --- |
|  |  |  | **During animal birth assistance ^1^** | | | **During daily farm activities^2^** | | | **Farm biosecurity adherence** | | | **Overall combined all domains** | | |
|  |  |  | **Unfavorable** | **Favorable** |  | **Unfavorable** | **Favorable** |  | **Unfavorable** | **Favorable** |  | **Unfavorable** | **Favorable** |  |
|  | **(N=315)** | | **(n=213)** | **(n=102)** |  | **(n=99)** | **(n=216)** |  | **(n=147)** | **(n=168)** |  | **(n=252)** | **(n=63)** |  |
|  | **n (%)** | | **n (%)** | **n (%)** | **p-value** | **n (%)** | **n (%)** | **p-value** | **n (%)** | **n (%)** | **p-value** | **n (%)** | **n (%)** | **p-value** |
| **Perceived occupational susceptibility to brucellosis** | |  |  |  |  |  |  |  |  |  |  |  |  |  |
| *Host range and zoonotic susceptibility* |  | |  |  |  |  |  |  |  |  |  |  |  |  |
| Ruminant farmers have a higher risk of contracting brucellosis than people in other occupations. | 115 (36.5) | | 72 (33.8) | 43 (42.2) | 0.188 | 32 (32.3) | 83 (38.4) | 0.358 | 41 (27.9) | 74 (44) | 0.004 | 84 (33.3) | 31 (49.2) | 0.028 |
| Other livestock farmers, such as those raising pigs, have a higher risk of contracting brucellosis than people in other occupations. | 114 (36.2) | | 77 (36.2) | 37 (36.3) | >0.999 | 35 (35.4) | 79 (36.6) | 0.934 | 48 (32.7) | 66 (39.3) | 0.269 | 88 (34.9) | 26 (41.3) | 0.429 |
| People in non-animal-related professions do not have the same risk level of contracting brucellosis as those who work directly with animals. | 215 (68.3) | | 154 (72.3) | 61 (59.8) | 0.036 | 70 (70.7) | 145 (67.1) | 0.615 | 109 (74.1) | 106 (63.1) | 0.048 | 176 (69.8) | 39 (61.9) | 0.290 |
| Overall perceived susceptibility attitude, positive^b^ | 33 (10.5) | | 20 (9.4) | 13 (12.7) | 0.476 | 7 (7.1) | 26 (12.0) | 0.255 | 9 (6.1) | 24 (14.3) | 0.030 | 22 (8.7) | 11 (17.5) | 0.073 |
| **Perceived disease impact and severity** |  | |  |  |  |  |  |  |  |  |  |  |  |  |
| *Disease impact and severity* |  | |  |  |  |  |  |  |  |  |  |  |  |  |
| Brucellosis has an impact on the economy and health of both humans and animals. | 236 (74.9) | | 158 (74.2) | 78 (76.5) | 0.764 | 72 (72.7) | 164 (75.9) | 0.640 | 110 (74.8) | 126 (75.0) | >0.999 | 187 (74.2) | 49 (77.8) | 0.673 |
| Brucellosis is a disease that needs to be prioritized for control and prevention. | 241 (76.5) | | 159 (74.6) | 82 (80.4) | 0.325 | 71 (71.7) | 170 (78.7) | 0.224 | 109 (74.1) | 132 (78.6) | 0.429 | 190 (75.4) | 51 (81.0) | 0.445 |
| Overall perceived disease impact and severity, positive^b^ | 212 (67.3) | | 142 (66.7) | 70 (68.6) | 0.827 | 66 (66.7) | 146 (67.6) | 0.973 | 96 (65.3) | 116 (69) | 0.558 | 168 (66.7) | 44 (69.8) | 0.741 |
| **Attitude toward prevention and control** |  | |  |  |  |  |  |  |  |  |  |  |  |  |
| *Attitude toward prevention of foodborne transmission* |  | |  |  |  |  |  |  |  |  |  |  |  |  |
| It is necessary to boil milk before consumption to reduce the risk of brucellosis. | 224 (71.1) | | 150 (70.4) | 74 (72.5) | 0.797 | 68 (68.7) | 156 (72.2) | 0.611 | 106 (72.1) | 118 (70.2) | 0.810 | 176 (69.8) | 48 (76.2) | 0.401 |
| *Personal protective measures and hygiene* |  | |  |  |  |  |  |  |  |  |  |  |  |  |
| Washing hands after contacting animals is not troublesome. | 234 (74.3) | | 151 (70.9) | 83 (81.4) | 0.064 | 65 (65.7) | 169 (78.2) | 0.026 | 108 (73.5) | 126 (75.0) | 0.856 | 179 (71) | 55 (87.3) | 0.013 |
| Using personal protective equipment, such as boots and gloves, is necessary when working with farm animals. | 237 (75.2) | | 154 (72.3) | 83 (81.4) | 0.108 | 66 (66.7) | 171 (79.2) | 0.025 | 111 (75.5) | 126 (75.0) | >0.999 | 186 (73.8) | 51 (81.0) | 0.312 |
| Overall personal prevention attitude, positive^b^ | 168 (53.3) | | 108 (50.7) | 60 (58.8) | 0.218 | 44 (44.4) | 124 (57.4) | 0.043 | 76 (51.7) | 92 (54.8) | 0.667 | 130 (51.6) | 38 (60.3) | 0.271 |
| *Farm biosecurity* |  | |  |  |  |  |  |  |  |  |  |  |  |  |
| Using disinfectant on the farm is necessary. | 208 (66.0) | | 137 (64.3) | 71 (69.6) | 0.424 | 64 (64.6) | 144 (66.7) | 0.823 | 98 (66.7) | 110 (65.5) | 0.918 | 163 (64.7) | 45 (71.4) | 0.388 |
| New animals purchased or brought into the farm may carry brucellosis and potentially transmit it to the existing herd. | 146 (46.3) | | 88 (41.3) | 58 (56.9) | 0.014 | 38 (38.4) | 108 (50) | 0.072 | 54 (36.7) | 92 (54.8) | 0.002 | 108 (42.9) | 38 (60.3) | 0.019 |
| New animals brought onto the farm should not be purchased from any farm without knowing their history. | 204 (64.8) | | 136 (63.8) | 68 (66.7) | 0.716 | 65 (65.7) | 139 (64.4) | 0.922 | 97 (66.0) | 107 (63.7) | 0.759 | 161 (63.9) | 43 (68.3) | 0.616 |
| It is necessary to notify a veterinarian or Department of Livestock officers if farm animals are sick and suspected of having brucellosis. | 256 (81.3) | | 173 (81.2) | 83 (81.4) | >0.999 | 78 (78.8) | 178 (82.4) | 0.543 | 121 (82.3) | 135 (80.4) | 0.765 | 205 (81.3) | 51 (81.0) | >0.999 |
| Overall farm-level prevention attitude, positive^b^ | 78 (24.8) | | 46 (21.6) | 32 (31.4) | 0.082 | 19 (19.2) | 59 (27.3) | 0.159 | 33 (22.4) | 45 (26.8) | 0.448 | 61 (24.2) | 17 (27.0) | 0.769 |
| Overall combined attitude toward prevention of brucellosis at personal and farm-level, positive^b^ | 57 (18.1) | | 35 (16.4) | 22 (21.6) | 0.341 | 13 (13.1) | 44 (20.4) | 0.164 | 24 (16.3) | 33 (19.6) | 0.538 | 46 (18.3) | 11 (17.5) | >0.999 |
| **Facilitators to animal brucellosis screening** |  | |  |  |  |  |  |  |  |  |  |  |  |  |
| *Disease screening* |  | |  |  |  |  |  |  |  |  |  |  |  |  |
| Testing animals on the farm for brucellosis is not a waste of labor. | 179 (56.8) | | 126 (59.2) | 53 (52.0) | 0.278 | 58 (58.6) | 121 (56.0) | 0.761 | 94 (63.9) | 85 (50.6) | 0.023 | 149 (59.1) | 30 (47.6) | 0.132 |
| Screening animals on the farm for brucellosis is not a waste of time. | 177 (56.2) | | 122 (57.3) | 55 (53.9) | 0.660 | 58 (58.6) | 119 (55.1) | 0.647 | 93 (63.3) | 84 (50.0) | 0.024 | 146 (57.9) | 31 (49.2) | 0.268 |
| Screening animals on the farm for brucellosis is not a waste of money. | 168 (53.3) | | 114 (53.5) | 54 (52.9) | >0.999 | 53 (53.5) | 115 (53.2) | >0.999 | 89 (60.5) | 79 (47.0) | 0.022 | 137 (54.4) | 31 (49.2) | 0.553 |
| Farmers are willing to cooperate if free brucellosis testing services are provided. (Strategy to reduce barrier) | 273 (86.7) | | 180 (84.5) | 93 (91.2) | 0.146 | 78 (78.8) | 195 (90.3) | 0.009 | 131 (89.1) | 142 (84.5) | 0.303 | 215 (85.3) | 58 (92.1) | 0.229 |
| Overall facilitators attitude, positive^b^ | 127 (40.3) | | 88 (41.3) | 39 (38.2) | 0.690 | 38 (38.4) | 89 (41.2) | 0.726 | 71 (48.3) | 56 (33.3) | 0.01 | 105 (41.7) | 22 (34.9) | 0.405 |
| **Overall combined attitude toward occupational brucellosis, positive^b^** | **5 (1.6)** | | **4 (1.9)** | **1 (1.0)** | **>0.999^F^** | **1 (1)** | **4 (1.9)** | **>0.999F** | **1 (0.7)** | **4 (2.4)** | **0.377^F^** | **4 (1.6)** | **1 (1.6)** | **>0.999^F^** |

^1^ Only farmers reporting animal birth assistance activities were included. Favorable personal preventive practice was defined as simultaneous adoption of both glove use and boot use during birth assistance.

^2^ Favorable practices reflected farmers who reported both regular use of boots during farm work and personal hygiene after farm activity; included all respondents (n=385) engaged in daily farm operations.

^a^ p-values by Chi-square test unless otherwise indicated.

^F^ p-values by Fisher’s exact test.

^b^ Positive attitude was defined as the combination of participants who responded "strongly agree" or "agree" to each statement.
