# Supplementary material for: Occupational brucellosis among livestock farmers in Northeastern Thailand: A cross-sectional study of knowledge, attitudes, and practices from a One Health perspective
Source: PLoS Negl Trop Dis. 2026 Oct 27;20(10):e0014788. doi: 10.1371/journal.pntd.0014788 (PMC13712670; doi:10.1371/journal.pntd.0014788)
Supplement: Supplementary file 8 [file pntd.0014788.s009.docx]

|  | **Introduction** |
| --- | --- |
| 77-78 | Please provide some numbers, e.g. what the incidence is or what proportion of at-risk populations have been found to be exposed, or if global burden estimates exist. |
| 89 | Please provide information on whether evidence on the effectiveness of prevention exists and briefly list the main findings from the other study published from Thailand that is referred to and the rationale for carrying out another survey (overcoming some limitations of the previous one?).  Also, something may have gone wrong with the reference numbering: reference 4 is about KAP in Thailand but in the main manuscript reference 5 is listed, please check other references to make sure they are correct. |
|  | Is there any evidence linking favourable practice to lower exposure? If there is, please refer to it in the introduction; if there is not, I would suggest mentioning it in the section on potential future studies |
| Methods | |
| 114 | I’m not sure I correctly understand the sample size calculation: This was based on correctly identifying practices practiced by 24% of the sampled population? |
| 119 | “Participants were recruited through coordination with provincial livestock officers”. Please specify which criteria were used, if participants were selected randomly from a list or purposively, if it was decided to include the same proportion of participants across the three provinces or across cattle types |
|  | It appears that all variables collected were included in the multivariable model, adjusted estimates therefore have large confidence interval, I would recommend including in the multivariable model only variables with p-values <0.1 in univariate analysis to avoid overdispersion, this would increase the statistical power of the multivariable analysis. This remains of course a choice the authors should make. |
| Results | |
|  | Were all farmers from different farms or were they clustered? Could you provide this information? E.g.: There wore 385 individuals from xx farms. If they were clustered, a clustered analysis may be more appropriate, if it is not known whether some were from the same farm, it should be listed in the limitations. |
|  | In the limitations section it is listed that some participants had missing information and were excluded, it would be useful to include at the beginning of the results section how many farmers were invited, how many agreed to participate, and how many were included in the final analysis, this could be presented in text or as a flowchart. |
| 235 | What is k6b? k6c? I imagine this is the number of each question, I would remove it |
| 244 | You refer to “comprehensive adherence”, does this mean adherence to all recommended practices? I think it would be useful to include this definition in the methods. |
|  | As above, I would recommend removing the codes of the questions |
| 274-275 | Please explain in the methods what the definitions for “prevention-focused attitude” and “combined preventive practices” was |
| 299-300 | “Knowledge of host range”… “and clinical manifestation”… . The Confidence intervals of the odds ratios for these associations are well below one; I would mention that the findings were not statistically significant. |
|  | **Discussion** |
| 418 | Probable typo, I think you are referring to Germany here |
|  | Would the favourable behaviours promoted for Brucella prevent farmers from other risks too? E.g. Anthrax or bovine tuberculosis? I think it would be valuable to mention. |
|  | **Tables** |
|  | In table 2 315 subjects are listed in the “practice” columns, please add a note to the legend explaining the reason for the discrepancy. Also, in Table 3, the multivariable regression includes 385 subjects (not 315), please explain in the methods if missing values imputed and how. If I understand correctly, in the methods it is stated that they were excluded, if they were excluded, please amend Table 3 as appropriate. |
