# Supplementary material for: Occupational brucellosis among livestock farmers in Northeastern Thailand: A cross-sectional study of knowledge, attitudes, and practices from a One Health perspective
Source: PLoS Negl Trop Dis. 2026 Oct 27;20(10):e0014788. doi: 10.1371/journal.pntd.0014788 (PMC13712670; doi:10.1371/journal.pntd.0014788)
Supplement: Supplementary file 9 [file pntd.0014788.s011.docx]

**Point-by-Point Response to Reviewers**

*Manuscript ID PNTD-D-26-00283 entitled "Occupational brucellosis among livestock farmers in Northeastern Thailand: a cross-sectional study of knowledge, attitudes, and practices from a One Health perspective"*

Dear Editors and Reviewers,

We sincerely thank the Editor and reviewers for their thorough and constructive evaluation of our manuscript. We have carefully considered each comment and revised the manuscript accordingly. All revisions are indicated in the marked-up copy of the manuscript labeled "Revised Manuscript with Track Changes." An unmarked version is provided separately as "Manuscript." A point-by-point response to each journal requirement and reviewer comment is provided below.

# **Journal Requirements**

**Requirement 1:** Please ensure that the CRediT author contributions listed for every co-author are completed accurately and in full. At this stage, the following Authors/Authors require contributions: Savek Kiatsomphob, Karunasinee Suphaphiphat, Bopit Puyati, and Kan Kledmanee. Please ensure that the full contributions of each author are acknowledged in the "Add/Edit/Remove Authors" section of our submission form. The list of CRediT author contributions may be found here: https://journals.plos.org/plosntds/s/authorship#loc-author-contributions

**Response:** We thank the editorial office for this requirement. We have updated the CRediT author contributions for all co-authors in the "Add/Edit/Remove Authors" section of the submission form. The contributions are now completed accurately and in full as follows:

- **Savek Kiatsomphob:** Conceptualization, Methodology, Validation, Writing – original draft, Writing – review & editing
- **Karunasinee Suphaphiphat:** Conceptualization, Methodology, Validation, Formal analysis, Writing – review & editing
- **Bopit Puyati:** Investigation, Resources, Supervision, Visualization, Writing – review & editing
- **Kan Kledmanee:** Conceptualization, Data curation, Formal analysis, Funding acquisition, Project administration, Validation, Visualization, Writing – original draft, Writing – review & editing

These contributions are consistent with the CRediT author contribution statement reported in the revised manuscript [Lines 647-654] and are in accordance with the journal guidelines.

**Requirement 2:** Your manuscript's sections are not in the correct order. Please amend to the following order: Abstract, Introduction, Results, Discussion, and Methods

**Response:** We thank the editorial office for this requirement. The Methods section has been relocated to follow the Discussion section, in accordance with the journal's required manuscript structure. The reordered sections are reflected in the tracked-changes version of the manuscript. The reference numbering is reordered in this revised version [Lines 677-869].

**Requirement 3:** Tables should not be uploaded as individual files. Please remove these files and include the Tables in your manuscript file as editable, cell-based objects. For more information about how to format tables, see our guidelines: https://journals.plos.org/plosntds/s/tables

**Response:** We thank the editorial office for this requirement. All tables have been removed from individual files and incorporated into the manuscript as editable, cell-based objects using the Word table tool. Each table has been placed directly after the paragraph in which it is first cited, in accordance with PLOS Neglected Tropical Diseases' formatting guidelines.

- Table 1 appears at Lines 624-625
- Table 2 appears at Lines 156-164
- Table 3 appears at Lines 237-247

**Requirement 4:** We have noticed that you have uploaded Supporting Information files, but you have not included a list of legends. Please add a full list of legends for your Supporting Information files after the references list.

**Response:** We thank the editorial office for this requirement. A full list of legends for all Supporting Information files has been added to the manuscript immediately after the References section, in accordance with PLOS Neglected Tropical Diseases' formatting guidelines. The legends appear at Lines 870-884

- **S1 Questionnaire.** KAP questionnaire on brucellosis in livestock farmers in Northeastern Thailand (Lines 872-873)
- **S2 Fig.** Response scale visual guide for KAP questionnaire (Lines 874)
- **S3 Table.** Awareness and knowledge regarding occupational brucellosis among ruminant farmers (N=385) (Lines 875-876)
- **S4 Table.** Positive attitude regarding occupational brucellosis among ruminant farmers (N=385) (Lines 877-878)
- **S5 Table.** Favorable preventive practices for occupational brucellosis prevention and control among ruminant farmers (Lines 879-880)
- **S6 Table.** Awareness and satisfactory knowledge regarding favorable preventive practices for occupational brucellosis among ruminant farmers (N=315) (Lines 881-882)
- **S7 Table.** Positive attitude regarding favorable preventive practices for occupational brucellosis among ruminant farmers (N=315) (Lines 883-884)
- **S8 File.** Available data (Lines 885)

**Requirement 5:** Potential Copyright Issues on (i) Striking image, and (ii) Figure 1

**Requirement 6:** Financial Disclosure statement.

**Requirement 7:** Inconsistency information about (iii) Financial Disclosure

**Response:** We thank the editorial office for the clarification on these requirements, as communicated via email on June 7, 2026.

**Regarding (i) Striking Image:** We confirm that the Striking Image was photographed by the corresponding author (Kan Kledmanee) using a personal mobile device during fieldwork. We are the sole copyright holder and hereby grant permission to publish this image under the CC BY 4.0 license. A Word document containing the image caption, credit line, and explicit confirmation that the Striking Image may be published under the Creative Commons Attribution License (CC BY 4.0) has been uploaded alongside the Striking Image file in the resubmission portal, upload it as "Other" file.

**Regarding (ii) Figure 1:** We thank the editorial office for confirming that DIVA-GIS data is compatible with the CC BY 4.0 license. A reference to DIVA-GIS (https://www.diva-gis.org) as the source of the administrative boundary shapefile has been added to the figure legend of **Figure 1** in the revised manuscript (Line 609-610).

**Regarding (iii) Financial Disclosure:** The Financial Disclosure statement has been corrected in the revised manuscript to accurately reflect the funding received for this study. We apologize for the inconsistency between the Financial Disclosure statement and the submission form. The corrected statement reads as follows:

"This research project has been supported by Mahidol University (Fundamental Fund: fiscal year 2025 by National Science Research and Innovation Fund (NSRF); grant number FF-173/2568 to KK). The funders had no role in study design, data collection and analysis, decision to publish, or preparation of the manuscript."

The updated statement appears at Lines 656-660.

**Other Comment from Editorial/General:**

References should be checked overall — some numbers appear to have shifted from the adequate location (links may require refreshing). There are also some minor grammatical errors throughout the manuscript.

**Response:** We thank the reviewer for these observations. We have carefully reviewed and corrected the reference numbering throughout the manuscript to ensure each citation appears in the appropriate location. All reference links have been refreshed and verified. In addition, the manuscript has been thoroughly reviewed for grammatical accuracy, and minor errors identified throughout the text have been corrected in the revised version.

**Reviewer 1 (R1)**

We thank Reviewer 1 for their thorough and constructive review. The comments have helped us strengthen the epidemiological framing, clarify the study's contribution, and improve the transparency of our analytical approach.

**R1 Comment 1 for *Methods* Section: Epidemiological context of human brucellosis**

The introduction mainly described the prevalence of brucellosis in animals but does not provide sufficient information on the epidemiology of human brucellosis in Thailand. Since brucellosis is a notifiable zoonotic disease under the Communicable Disease Act, the authors should include information on incidence or reported case numbers of human brucellosis in Thailand, trends of human cases over time and occupational groups most at risk. Providing this epidemiological background would strengthen the justification for studying preventive behaviors among livestock farmers.

**Response:** We thank the reviewer for this valuable suggestion. We agree that the epidemiological context of human brucellosis in Thailand was insufficiently presented in the original manuscript. We have therefore substantially revised the **Introduction** [Lines 80-93] to (i) explicitly note the notifiable status of brucellosis under Thailand’s Communicable Diseases Act, (ii) summarize national surveillance evidence indicating a sustained accumulation of human brucellosis cases over the past two decades, and (iii) highlight epidemiological investigations documenting clustered infections among goat keepers and seropositivity in vulnerable clinical cohorts, such as pregnant women with adverse pregnancy outcomes. Together, these additions provide a clearer picture of temporal trends and high‑risk occupational and community groups, thereby strengthening the public health rationale for focusing on preventive behaviors among livestock farmers in this setting.

**R1 Comment 2 for *Methods* Section: Justification for conducting another KAP study**

The manuscript stated that there was limited information regarding farmers’ KAP on brucellosis in Thailand. However, some previous studies have already investigated KAP among livestock farmers, particularly goat farmers. The authors should clarify the added value of this study. Clearly articulating the knowledge gap addressed by this study will strengthen the manuscript’s contribution.

**Response:** We thank the reviewer for this important observation. We acknowledge that prior KAP studies on brucellosis among livestock farmers in Thailand exist, notably among goat farmers. We have revised the **Introduction** [Lines 122-124] to clarify that the present study adds value by focusing on mixed‑ruminant farmers (cattle, buffalo, and goats) in Northeastern Thailand and by examining broader contextual and system‑level factors that may influence preventive behaviours, which have not been considered in previous KAP studies in Thailand.

**R1 Comment 3 for *Methods* Section: Sample size calculation and statistical analysis**

The sample size was calculated using a single population proportion formula based on the prevalence of glove use. However, the analysis includes comparative analyses and multivariable logistic regression across several subgroups, such as farm certification status and livestock ownership. Because the sample size was not calculated specifically for comparative analyses, the statistical power for detecting differences between groups may be limited. The authors should therefore acknowledge this issue in the limitations section and clarify that subgroup analyses may have reduced statistical power.

**Response:** We thank the reviewer for raising this methodological concern. We fully agree that the original sample size calculation, based on a single population proportion for glove use prevalence, was not designed to ensure adequate statistical power for comparative subgroup analyses or multivariable logistic regression. In the revised **Statistical analysis** [Lines 626-633], we now explicitly clarify that multivariable models were fitted separately for each outcome domain and that the combined preventive practice outcome was analysed in a prespecified high-exposure subgroup of farmers who reported assisting animal births within the previous year (N = 315), because birth-related practice items were only applicable to this subgroup. We have also added a statement to the **Strengths and limitations** section [Lines 455-461] noting that subgroup analyses, particularly those involving certified farms and small ruminant ownership, may have reduced statistical power and that the corresponding estimates should be interpreted with caution.

**R1 Comment 4 for *Methods* Section: Potential confounding factors in the association between age and knowledge**

The results suggested that older farmers demonstrated higher knowledge regarding brucellosis prevention. However, age may be associated with other factors such as education level, years of farming experience, or exposure to previous livestock disease control campaigns. It should be clarified whether these variables were included in the adjusted models. The authors should explain whether the association between age and knowledge remained significant after adjusting for these potential confounders.

**Response:** We thank the reviewer for this important observation. We agree that age may be correlated with education, years of farming experience, and prior exposure to disease‑control activities. In our multivariable model for satisfactory knowledge, we adjusted simultaneously for age group, education level, years of farming experience, primary income source, and ruminant species ownership (Table 3). After adjustment, farmers aged over 60 years had significantly higher odds of satisfactory knowledge compared with those aged 40 years or younger (aOR 2.65, 95% CI 1.06–6.62), whereas years of farming experience did not show an independent association with knowledge. We did not, however, collect specific data on previous participation in livestock disease‑control campaigns, and older farmers may therefore have had more frequent contact with veterinary extension or disease‑control activities over time, which may also help explain the observed age-knowledge pattern. In response to this comment, we now clarify the adjusted model specification in the **Results** [Lines 234-236], **Table 3** [Lines 237-247], and explicitly note in the **Discussion** [Lines 305-313] and **Strengths and limitations** [Lines 451-461].

**R1 Comment 5 for *Results* Section: Nationality of farmers and the role of migrant workers**

The manuscript did not clearly describe the nationality of study participants. Since migrant workers are frequently involved in livestock farming in Thailand and the study reported that no respondents consumed raw milk or placenta, the authors should clarify: (1) whether all participants were Thai nationals, (2) whether migrant workers were included in the study population, and (3) how this might influence interpretation of results and generalizability.

**Response:** We thank the reviewer for raising this important consideration. We would like to clarify that all study participants were Thai nationals, as recruitment was conducted exclusively through the DLD‑registered farmer network. Registration with the DLD requires Thai national identification; therefore, the study population comprised Thai nationals only, and migrant workers were not captured within this sampling frame. This has been clarified in the revised **Sample size and participant recruitment** subsection [Lines 550-551]. We acknowledge that this sampling approach may limit generalizability to unregistered or migrant farm workers, who may have distinct risk profiles and preventive practices. This limitation has been noted in the revised **Strengths and limitations** [Lines 467-471].

**R1 Comment 6 for *Conclusions* Section: Role of community-based health actors**

In the implications section, the authors recommended delivering training through Village Health Volunteers. However, the role of these volunteers is not described earlier in the manuscript. To strengthen this recommendation, the authors should briefly discuss the role of Village Health Volunteers in health education and community surveillance in Thailand and their potential role in zoonotic disease prevention. In addition, the authors may consider mentioning livestock volunteers under the Department of Livestock Development, who play important roles in animal disease reporting and farmer education. Including both actors would better reflect the operational One Health collaboration at the community level.

**Response:** We thank the reviewer for this constructive suggestion. We agree that our recommendation to engage Village Health Volunteers (VHVs) required clearer contextual grounding. In the revised **Implications** section [Lines 496-504], we now describe the established role of VHVs in community health education and local syndromic surveillance in Thailand, and then explicitly position them as key facilitators for delivering occupational brucellosis training to livestock-keeping households. In addition, we have incorporated Livestock Volunteers (LVs) under the Department of Livestock Development (DLD), highlighting their responsibilities in village‑level herd surveillance, animal disease reporting, and farmer training. Together, these twin volunteer networks exemplify an operational, community‑level One Health platform that can be leveraged to co-deliver integrated human–animal biosecurity messages to smallholder farmers.

**R1 Comment 7 for *Conclusions* Section: Generalizability**

The generalizability of the findings beyond the three provinces studied should be briefly discussed.

**Response:** We thank the reviewer for this insightful comment. We have revised the **Strength and Limitation** section [Lines 467-471] to explicitly address the generalizability of our findings. While the selected provinces represent areas of high livestock productivity, we have now clarified that our reliance on the DLD‑registered farmer network restricted the sample to Thai nationals. Consequently, the findings may not be fully generalizable to other regions with different farming systems, unregistered farmers, or migrant agricultural workers.

**Reviewer 2 (R2)**

We thank Reviewer 2 for their thorough and detailed review. The comments spanning the Introduction, Methods, Results, Discussion, Tables, and Figures have helped us considerably improve the clarity, methodological transparency, and overall quality of the manuscript. We have addressed each point individually below.

**R2 Comment 1 for *Methods* Section:**

I recommended the authors use a different approach for the multivariable analysis to increase statistical power, this decision should rest on the authors but should be argumented, some definitions should also be added; more details and other minor recommendations are included in the attachment.

**Response:** We thank the reviewer for this overarching methodological comment. We decided to retain a prespecified full multivariable model for each outcome rather than apply p‑value–based variable selection, because this approach is less prone to selection bias and provides more valid adjusted estimates when potential confounders are defined a priori. We have revised the **Statistical analysis** subsection [Lines 642-644] to clarify this rationale and to describe more explicitly how the binary KAP outcomes and analytic samples were defined for each model. **Table 1** in the Methods summarises the classification criteria for all binary KAP outcomes, including the combined prevention‑focused attitude and combined preventive practices. In addition, we have updated **the footnote of Table 3** [Lines 240-247] and **the legend of Fig 3** [Lines 265-272] in the Results to clearly indicate which models used all farmers (N=385) and which used the predefined high‑exposure subgroup (N=315).

The reviewer also included additional, more specific remarks in the attached table, which we reproduce and respond to below.

**R2 Comment 2 [Introduction, Lines 77–78]:**

Please provide some numbers, e.g. what the incidence is or what proportion of at-risk populations have been found to be exposed, or if global burden estimates exist.

Response: We thank the reviewer for this helpful suggestion. In the revised Introduction [Lines 80-87], we now provide quantitative information on human brucellosis in Thailand, including national surveillance evidence and an example of a goat‑farm–associated outbreak among exposed keepers, as well as seropositivity documented in vulnerable clinical cohorts such as pregnant women with adverse pregnancy outcomes. We have also added a brief sentence summarizing global estimates of human brucellosis burden to contextualize the national situation within the wider endemic distribution.

**R2 Comment 3 [Introduction, Line 89]:**

Please provide information on whether evidence on the effectiveness of prevention exists and briefly list the main findings from the other study published from Thailand that is referred to and the rationale for carrying out another survey (overcoming some limitations of the previous one?). Also, something may have gone wrong with the reference numbering: reference 4 is about KAP in Thailand but in the main manuscript reference 5 is listed, please check other references to make sure they are correct. ตรวจสอบ Reference (in-text citation no. vs. in reference list)

**Response:** We thank the reviewer for this helpful comment. In the revised **Introduction** [Lines 106-126], we now add evidences on brucellosis prevention, including seroprevalence and risk factor data that highlight the role of biosecurity and high‑risk husbandry practices. We also explicitly describe the key findings of the only published Thai KAP study, which focused on small-scale goat farmers and documented important gaps in brucellosis-specific knowledge and preventive behaviours and additional relevant citations to clarify the gap of knowledge and rationale of our KAP study.

We thank the reviewer for noting the reference mismatch. We have carefully checked and corrected the in-text citation numbering so that all references now align with the **reference list** [Lines 677-869].

**R2 Comment 4 [Introduction — no line specified]:**

Is there any evidence linking favourable practice to lower exposure? If there is, please refer to it in the introduction; if there is not, I would suggest mentioning it in the section on potential future studies

**Response**: We thank the reviewer for this valuable point. We have now added a concise summary of existing evidence linking preventive practices to lower exposure in the **Introduction** [Lines 118-120]. Specifically, we cite a systematic review and meta-analysis and occupational studies (Igawe *et al*., 2020; Pereira *et al*., 2020; Tiwari *et al*., 2022) showing that adequate biosecurity and consistent PPE use are associated with lower *Brucella* seroprevalence and reduced occupational exposure among high‑risk workers.

**R2 Comment 5 [Methods, Line 114]:**

I’m not sure I correctly understand the sample size calculation: This was based on correctly identifying practices practiced by 24% of the sampled population?

**Response:** We thank the reviewer for seeking clarification. The p = 0.24 was taken from the proportion of farmers reporting preventive glove use in a previous Thai KAP study (Peck *et al*., 2019), and was used as the expected prevalence in Cochran’s formula, as described in the **Sample size and participant recruitment** subsection [Lines 545-547]. We acknowledge, as also noted in our response to Reviewer 1 Comment 3, that this single‑proportion–based calculation did not specifically account for the power of subgroup or multivariable analyses, and we have therefore highlighted this as a limitation in the revised **Strength and limitation** section [Lines 455-461].

**R2 Comment 6 [Methods, Line 119]:**

“Participants were recruited through coordination with provincial livestock officers”. Please specify which criteria were used, if participants were selected randomly from a list or purposively, if it was decided to include the same proportion of participants across the three provinces or across cattle types.

**Response:** We thank the reviewer for this important methodological observation. As clarified in the revised **Sample size and participant recruitment** subsection [Lines 550-551], the study population comprised ruminant livestock farmers registered in the DLD database in the three provinces. Participants were recruited using non‑probability sampling through coordination with provincial livestock officers and routine extension activities; during these visits, eligible Thai farmers registered in the DLD database were invited to participate in the study. We did not apply formal random sampling from the registration lists, and no quotas were imposed for equal representation across provinces or for specific livestock species, as recruitment was constrained by routine service schedules and field feasibility.

**R2 Comment 7 [Methods — no line specified]:**

It appears that all variables collected were included in the multivariable model, adjusted estimates therefore have large confidence interval, I would recommend including in the multivariable model only variables with p-values <0.1 in univariate analysis to avoid overdispersion, this would increase the statistical power of the multivariable analysis. This remains of course a choice the authors should make.

**Response:** We appreciate the reviewer’s concern that including too many predictors could lead to imprecise adjusted estimates with wide confidence intervals. We would like to clarify that our multivariable models did not include all variables collected in the questionnaire. For each outcome, we therefore adjusted only for covariates prespecified a priori as epidemiologically relevant confounders, rather than selecting variables based on univariate p‑values. In line with recent simulation‑based work by Luijken *et al.* (2024), we retained this prespecified model structure to avoid selection bias and unstable adjusted estimates. We have clarified this rationale in the revised **Statistical analysis** subsection [Lines 642-644]. Also, we have revised the **footnote of** **Table 3** [Lines 240-247] and the legend for **Fig 3** [Lines 265-272] to make clear that the models were adjusted for all covariates presented in each table or panel, but not all variables collected in the questionnaire.

**R2 Comment 8 [Results — no line specified]:**

Were all farmers from different farms or were they clustered? Could you provide this information? E.g.: There wore 385 individuals from xx farms. If they were clustered, a clustered analysis may be more appropriate, if it is not known whether some were from the same farm, it should be listed in the limitations.

**Response:** We thank the reviewer for raising this point. In this study, each interview was conducted with the main ruminant livestock farmer in the household, and each registered farm household contributed only one farmer to the study. We have clarified this in the revised **Methods** section [Lines 554-556], so a clustered analysis was not required.

**R2 Comment 9 [Results — no line specified]:**

In the limitations section it is listed that some participants had missing information and were excluded, it would be useful to include at the beginning of the results section how many farmers were invited, how many agreed to participate, and how many were included in the final analysis, this could be presented in text or as a flowchart.

**Response:** We thank the reviewer for this helpful suggestion. In the revised **Results** section [Lines 140-141], we now report that 428 farmers were approached through the DLD‑registered farmer network, 415 agreed to participate, and 385 were included in the final analytical sample after excluding records with incomplete data. We opted to present this information in text at the beginning of the Results section, as it succinctly conveys the participant flow and aligns with the limitations already describing the exclusion of incomplete records.

**R2 Comment 10 [Results, Line 235]:**

What is k6b? k6c? I imagine this is the number of each question, I would remove it

**Response:** We thank the reviewer for identifying this typo. We have removed the “k6b” and “k6c” labels from the **Results** and other relevant sections in the revised manuscript [Lines 181-182].

**R2 Comment 11 [Results, Line 244]:**

You refer to “comprehensive adherence”, does this mean adherence to all recommended practices? I think it would be useful to include this definition in the methods.

**Response:** We thank the reviewer for this clarification request. In the revised **Methods** [Lines 249-263] and **Table 1**, we now state that *prevention-focused attitude* corresponds to the overall combined positive attitude toward occupational brucellosis, defined as positive only when all four attitude domains are favorable. We also clarify that combined preventive practices denote the *combined preventive practice outcome* in the predefined high-exposure subgroup (farmers assisting animal births; N = 315), which is classified as favorable only when all key personal- and farm-level practice items are favorable. This corresponds to *comprehensive adherence* to the recommended preventive practices, as the reviewer correctly inferred. These definitions are now used consistently in the **Results** and in **Fig.3B and 3C**.

**R2 Comment 12 [Results — no line specified]:**

As above, I would recommend removing the codes of the questions

**Response:** We thank the reviewer for this observation. In line with this suggestion and our response to *R2 Comment 9*, we have removed the question codes (“k6b”, “k6c” and similar item labels) from the **Results** text [Lines 201-202] in the revised manuscript and **Fig 2**.

**R2 Comment 13 [Results, Lines 274–275]:**

Please explain in the methods what the definitions for “prevention-focused attitude” and “combined preventive practices” was.

**Response:** We thank the reviewer for this clarification request. In the revised **Methods** [Lines 626-633] and Table 1, we now state that *prevention-focused attitude* corresponds to the overall combined positive attitude toward occupational brucellosis, defined as positive only when all four attitude domains are favorable. We also clarify that *combined preventive practices* denote the combined preventive practice outcome in the predefined high-exposure subgroup (farmers assisting animal births), which is classified as favorable only when all key personal- and farm-level practice items are favorable. These definitions are now used consistently in the Results and in **Fig. 3B and 3C**.

**R2 Comment 14 [Results, Lines 299–300]:**

“Knowledge of host range”… “and clinical manifestation”… . The Confidence intervals of the odds ratios for these associations are well below one; I would mention that the findings were not statistically significant.

**Response:** We thank the reviewer for this important clarification. In the revised **Results** [Lines 256-263], we have modified the text to state that knowledge of host range and zoonotic susceptibility, as well as knowledge of clinical manifestations in humans, showed positive but not statistically significant associations with a prevention‑focused attitude (Fig. 3B), in line with the confidence intervals crossing one. Because this issue also affected the interpretation of some related findings, we thoroughly reviewed the linked **Discussion** text for consistency and revised the *Cognitive and attitudinal drivers* subsection accordingly [Lines 367-370]. Additionally, we removed the discussion of awareness of the scientific term “brucellosis” from this subsection, as this point is more appropriately addressed under *Behavioral outcome and translation to practice* subsection, where it aligns with the borderline positive association observed for combined preventive practices. We are grateful to the reviewer for highlighting this issue, which has greatly helped us improve the precision and internal consistency of our manuscript.

**R2 Comment 15 [Discussion, Line 418]:**

Probable typo, I think you are referring to Germany here.

**Response:** We thank the reviewer for this careful reading of our manuscript. The cited study by Narimani *et al.* (2024) was conducted in Germi County in Ardabil Province, Iran. In the revised **Discussion** [Lines 383-385], we have simplified the study setting description to refer to “Iran,” which accurately reflects the location of the study and avoids potential confusion.

**R2 Comment 16 [Discussion — no line specified]:**

Would the favourable behaviours promoted for *Brucella* prevent farmers from other risks too? E.g. Anthrax or bovine tuberculosis? I think it would be valuable to mention.

**Response:** We thank the reviewer for this insightful suggestion. We agree that the favourable behaviours promoted for brucellosis prevention have important co‑benefits for other zoonoses. In the revised **Discussion** section [Lines 425-434], we now briefly note that consistent use of gloves and boots during parturition and careful handling of birth materials also provides cross‑protection against Q fever, which shares similar transmission pathways.

**R2 Comment 17 [Tables]:**

In table 2_315 subjects are listed in the “practice” columns, please add a note to the legend explaining the reason for the discrepancy. Also, in Table 3, the multivariable regression includes 385 subjects (not 315), please explain in the methods if missing values imputed and how. If I understand correctly, in the methods it is stated that they were excluded, if they were excluded, please amend Table 3 as appropriate.

**Response:** We thank the reviewer for carefully noting the apparent discrepancy in sample size between the overall study population (N = 385) and the subgroup of farmers included in the practice domain (N = 315). We confirm that this difference does not reflect missing data or imputation, but rather a predefined analytic restriction. As described in the revised **Statistical analysis** subsection [Lines 634-646], the combined preventive practice outcome was examined exclusively in a predefined high-exposure subgroup of farmers who reported assisting animal births within the previous year, because birth-related practice items were only applicable to this subgroup.

Consistent with this analytic approach, we have clarified in the **Results** section [Lines 187-204] that the practice-related findings are based on this predefined high-exposure subgroup of 315 farmers, and we now explicitly refer to them as “the predefined high-exposure subgroup” in the text. To indicate that knowledge and attitude outcomes are based on the full sample of 385 farmers, whereas combined preventive practices are based on the predefined high-exposure subgroup (N = 315), we have also revised **the title and footnote of Table 2 and 3** as follows:

- **Table 2 title** [Lines 156-157]: Sociodemographic characteristics and overall knowledge, attitude, and practice (KAP) levels regarding occupational brucellosis among ruminant farmers
- **Table 2 footnote** [Lines 163-164]:

^†^ Knowledge and attitude outcomes are based on the full sample of farmers (N = 385). Practice outcomes are based on the predefined high-exposure subgroup of farmers who reported assisting animal births within the previous year (N = 315).

- **Table 3 title** [Lines 237-238]: Multivariable logistic regression analysis of sociodemographic factors of satisfactory knowledge, prevention-focused attitude, and combined preventive practices towards brucellosis among ruminant farmers
- **Table 3 footnote** [Lines 246-247]:

^†^ Knowledge and attitude models include all farmers (N = 385). The combined preventive practice model is restricted to the predefined high-exposure subgroup only (farmers assisting animal births; N = 315).

**R2 Comment 18 for *Editorial and Data Presentation Modifications?***

See attachment, references should be checked (some numbers appear to have shifted from the adequate location, maybe the links require refreshing), there are some minor grammatical errors.

**Response:** We thank the reviewer for these observations. We have reviewed and corrected the reference numbering, refreshed the reference links, and amended minor grammatical errors throughout the revised manuscript.
